# Exploring radiation resistance-related genes in pancreatic cancer and their impact on patient prognosis and treatment

**DOI:** 10.3389/fimmu.2025.1524798

**Published:** 2025-03-03

**Authors:** Dong Dai, Sen Wang, Jiaze Li, Yu Zhao

**Affiliations:** ^1^ Department of Nuclear Medicine, Tianjin Cancer Hospital Airport Hospital, National Clinical Research Center for Cancer, Institute of Radiation Medicine, Chinese Academy of Medical Sciences and Peking Union Medical College, Tianjin, China; ^2^ Department of Molecular Imaging and Nuclear Medicine, Tianjin Medical University Cancer Institute and Hospital, National Clinical Research Center for Cancer, Key Laboratory of Cancer Prevention and Therapy, Tianjin’s Clinical Research Center for China, Tianjin, China; ^3^ Tianjin Key Laboratory of Radiation Medicine and Molecular Nuclear Medicine, Institute of Radiation Medicine, Chinese Academy of Medical Sciences and Peking Union Medical College, Tianjin, China

**Keywords:** pancreatic cancer, radiotherapy resistance, prognostic scoring model, immune microenvironment, personalized immunotherapy

## Abstract

**Background:**

Pancreatic cancer is a highly lethal disease with increasing incidence worldwide. Despite surgical resection being the main curative option, only a small percentage of patients are eligible for surgery. Radiotherapy, often combined with chemotherapy, remains a critical treatment, especially for locally advanced cases. However, pancreatic cancer’s aggressiveness and partial radio resistance lead to frequent local recurrence. Understanding the mechanisms of radiotherapy resistance is crucial to improving patient outcomes.

**Methods:**

Pancreatic cancer related gene microarray data were downloaded from GEO database to analyze differentially expressed genes before and after radiotherapy using GEO2R online tool. The obtained differentially expressed genes were enriched by GO and KEGG to reveal their biological functions. Key genes were screened by univariate and multivariate Cox regression analysis, and a risk scoring model was constructed, and patients were divided into high-risk group and low-risk group. Subsequently, Kaplan-Meier survival analysis was used to compare the survival differences between the two groups of patients, further analyze the differential genes of the two groups of patients, and evaluate their sensitivity to different drugs.

**Results:**

Our model identified 10 genes associated with overall survival (OS) in pancreatic cancer. Based on risk scores, patients were categorized into high- and low-risk groups, with significantly different survival outcomes and immune profile characteristics. High-risk patients showed increased expression of pro-inflammatory immune markers and increased sensitivity to specific chemotherapy agents, while low-risk patients had higher expression of immune checkpoints (CD274 and CTLA4), indicating potential sensitivity to targeted immunotherapies. Cross-dataset validation yielded consistent AUC values above 0.77, confirming model stability and predictive accuracy.

**Conclusion:**

This study provides a scoring model to predict radiotherapy resistance and prognosis in pancreatic cancer, with potential clinical application for patient stratification. The identified immune profiles and drug sensitivity variations between risk groups highlight opportunities for personalized treatment strategies, contributing to improved management and survival outcomes in pancreatic cancer.

## Introduction

Pancreatic cancer is a particularly aggressive cancer, with its incidence and mortality rates steadily increasing globally (1, 2). According to the statistics of China’s National Cancer Center in 2021, pancreatic cancer ranks 7th in the incidence of malignant tumors in men and 11th in women in China, and accounts for 6th in the malignancy related mortality (3). According to the 2023 Cancer Statistics Report, the death rate of pancreatic cancer will rank second only to lung cancer by 2030 (4).

PAAD (pancreatic adenocarcinoma) accounts for the vast majority (85-90%) of all pancreatic cancers, and TCGA’s PAAD program is almost entirely PDAC (pancreatic ductal adenocarcinoma) cases. Despite advances in therapeutic strategies, PAAD remains challenging to treat, primarily due to its late diagnosis, limited resect ability, and high resistance to conventional treatments such as radiotherapy (RT) (5). Surgical resection is the main method for pancreatic cancer patients to obtain cure and prolong survival time. Unfortunately, only a minority (15-20%) of patients can be surgically removed (6).

RT is an important treatment for pancreatic cancer, especially in combination with chemotherapy, which is the preferred treatment for locally advanced pancreatic cancer (7). Yet, PAAD’s intrinsic resistance to radiation and tendency for local recurrence limit the overall effectiveness of RT in improving patient outcomes (8, 9). This resistance, often coupled with a pro-inflammatory tumor microenvironment, facilitates tumor progression and immune escape, ultimately making PAAD more challenging to treat (10). Therefore, understanding the mechanisms of RT resistance and the dynamics of the immune microenvironment is essential for developing more targeted therapies.

To address these challenges, recent studies have focused on identifying specific gene expression profiles and immune cell infiltration patterns associated with RT resistance and immune evasion in PAAD. Through these investigations, researchers aim to stratify patients into high- and low-risk groups, thereby enabling more personalized treatment approaches. Genes that exhibit differential expression in response to RT have shown promise as predictive markers, offering insight into patient prognosis and potential therapeutic targets.

In this study, we utilized two independent GEO datasets (GSE179351 and GSE225767) to identify genes associated with RT resistance in PAAD and constructed a prognostic scoring model validated across multiple datasets. Additionally, we explored the differences in drug sensitivity between these risk groups, offering valuable insights for individualized treatment strategies. This study provides a comprehensive framework for understanding the immune and molecular landscape of PAAD, contributing to the broader goal of personalized therapy. By integrating gene expression, immune cell profiles, and drug response data, our findings support the development of more targeted, effective treatments for PAAD, ultimately aiming to improve patient survival and quality of life. Future research should focus on validating these biomarkers in larger cohorts and exploring immune-related predictive markers to further refine therapeutic strategies in PAAD.

## Materials and methods

### Data collection

To obtain the data needed for this study, we used Gene Expression Omnibus (GEO), a public functional genome database. We used two words “pancreatic cancer” and “radiation” as the keywords to obtain the required items, and finally selected the series GSE179351 and GSE225767 as the data set for our main analysis.

To obtain patient gene expression and clinical data, we included cases from The Cancer Genome Atlas (TCGA) project. We collected gene expression data from PAAD cases, including 179 tumor samples and 4 normal samples. All TCGA data, mRNA expression and clinical details were manually downloaded from the website and collated by R software.

### Analysis of differentially expressed genes

GEO2R was used to identify differentially expressed genes in the geo data sets GSE179351 and GSE225767 after RT compared to before RT. The differentially expressed genes (DEGs) in PAAD samples in TCGA database were identified by R package “edgeR”. Using |log_2_FC| ≥1 and *p*-value <0.05 as selection criteria, the range of DEGs was determined for further analysis. Volcano mapping using R software packages “limma” and “ggplot2”. Using gene annotation and analysis resources website “Metscape” (https://www.metascape.org/) for gene function analysis of enrichment.

### Cox regression analysis and prognostic model construction

After obtaining 121 co-up-regulated genes and 27 co-down-regulated genes between GSE179351 and GSE225767 as candidate genes for constructing radiation-resistant gene plates, we used univariate Cox regression to select the gene that was most correlated with OS (overall survival) in PAAD patients, and the *p*-value was set at 0.05.

Then, using the results of univariate Cox regression, we performed proportional risk regression analysis (multivariate Cox model) by the following formula.


Riskscore=∑ni=1(Coefficienti×xi)

Where, the risk score is the product of the mRNA expression of each key predictor gene (xi) and coefficient, which is derived from multivariate Cox regression analysis. Using the survival and survminer packages in R, 178 TCGA-PAAD patients were divided into high-risk group and low-risk group. On the basis of this grouping routine, survival differences between OS and risk scores were observed between the two groups. Logistic regression model was established by using R software package “glmnet”.

### Parameter setting and data set partitioning of random forest model

Random forest model was performed by R package ggRandomForests (v2.2.1). Regarding the allocation of the training and testing sets, 148 (83%) of samples to the training set and 30 (27%) to the testing set. In the parameter, there are 183 nodes and 454 edges contained inside this representation. Number of trees was set to 1000 and minimum size of terminal node was set to 10. Other parameters were default settings.

### Cell culture

PANC-1 cells were purchased from Wuhan Promoter Life Science & Technology Co., Ltd. (China). All cell lines were cultured at 37°C in an atmosphere of 5% CO_2_ using DMEM medium containing 10% fetal bovine serum (FBS) (Gibco) and 1% penicillin/streptomycin. When cell confluence reached 70%-80%, they were subjected to ionizing radiation treatment.

### Real-time qPCR

Trizol was used to extract RNA from cells. After RNA extraction, the quantity and quality of the RNA were analyzed using the Qnano spectrophotometry method from Yeasen. A reverse transcription reaction was performed using 1 μg of total RNA with Takara’s reverse transcription kit (RR047A). The resulting cDNA was subjected to qPCR analysis using Hieff UNICON^®^ Universal Blue qPCR SYBR Green Master Mix (Yeasen, Cat #11184ES08), and quantification was performed using Yeasen 80520ES03. In all experimental replicates, all expression levels were normalized to GAPDH. The primers used were as follows:

*ADAMTS12 Forward* (5′-CTTTGAAGGCGGCAACAGCAGA-3′)

*ADAMTS12 Reverse* (5′-TCTCACAGTCTGGCAGGAAGAG-3′)

*AKR1C2 Forward* (5′-CCGAAGCAAGATTGCAGATGGC-3′)

*AKR1C2 Reverse* (5′-TTTCAGTGACCTTTCCAAGGCTG-3′)

*ATP8B2 Forward* (5′-CGGCTATTCCTGCAAGATGCTG- 3′)

*ATP8B2 Reverse* (5′-GTCCTGATAGGTGAAGCCGTTG-3′)

*CCN4 Forward* (5′-AAGAGAGCCGCCTCTGCAACTT-3′)

*CCN4 Reverse* (5′-TCATGGATGCCTCTGGCTGGTA-3′)

*CTHRC1 Forward* (5′-CAGGACCTCTTCCCATTGAAGC-3′)

*CTHRC1 Reverse* (5′-GCAACATCCACTAATCCAGCACC-3′)

*GREM1 Forward* (5′-TCATCAACCGCTTCTGTTACGGC- 3′)

*GREM1 Reverse* (5′-CAGAAGGAGCAGGACTGAAAGG-3′)

*P3H3 Forward* (5′-CTGAGTGTCCTGCTCTTCTACC-3′)

*P3H3 Reverse* (5′-ATCGGAGGATGAAGCGCTGGAT-3′)

*PAPPA Forward* (5′-GGAACTGAAGAGAGTGAGCCATC-3′)

*PAPPA Reverse* (5′-CGTCGCATTGTTCACCTTGGTC-3′)

*POSTN Forward* (5′-CAGCAAACCACCTTCACGGATC- 3′)

*POSTN Reverse* (5′-TTAAGGAGGCGCTGAACCATGC-3′)

*TAFA2 Forward* (5′-GATCGGAAAGGATGGAGCTGTTC-3′)

*TAFA2 Reverse* (5′-GCGCATGTTCAATGTCATCAGCC-3′)

*GAPDH Forward* (5′-GGAGCGAGATCCCTCCAAAAT- 3′)

*GAPDH Reverse* (5′-GGCTGTTGTCATACTTCTCATGG-3′)

### Analysis of tumor microenvironment

Single sample gene set enrichment analysis (ssGSEA) was performed using R package “GSVA” to calculate the infiltration level of 28 immune cells. The immune gene sets were sourced from Charoentong’s study (11). Finally, we compared the expression levels of immune checkpoint molecules (CD274 and CTLA4) between the two groups of patients. TIDE was used to predict immunotherapy.

### Drug sensitivity

In this study, we explored the predictive value of risk score for immunotherapy and chemotherapy efficacy. Tumor immune dysfunction and rejection (TIDE) score (http://tide.dfci.harvard.edu/) is a kind of used to evaluate tumor immune escape mechanism in the immune microenvironment of tools. TIDE scores predict a patient’s response to immunotherapy, such as immune checkpoint inhibitors, by taking into account the ability of tumor cells to escape immune and the role of tumor-related immunosuppression. The drug was estimated using the “oncopredict” package in R to predict chemotherapy drug sensitivity in each patient.

### Statistical analysis

All statistical analysis and graphical visualizations were performed in R (version 4.3.2). Continuous variables were compared between groups using student t test or Wilcoxon rank sum test. *P*<0.05 was considered statistically significant (bilateral).

## Result

### Screening and functional analysis of genes related to RT resistance

**Figure 1:**
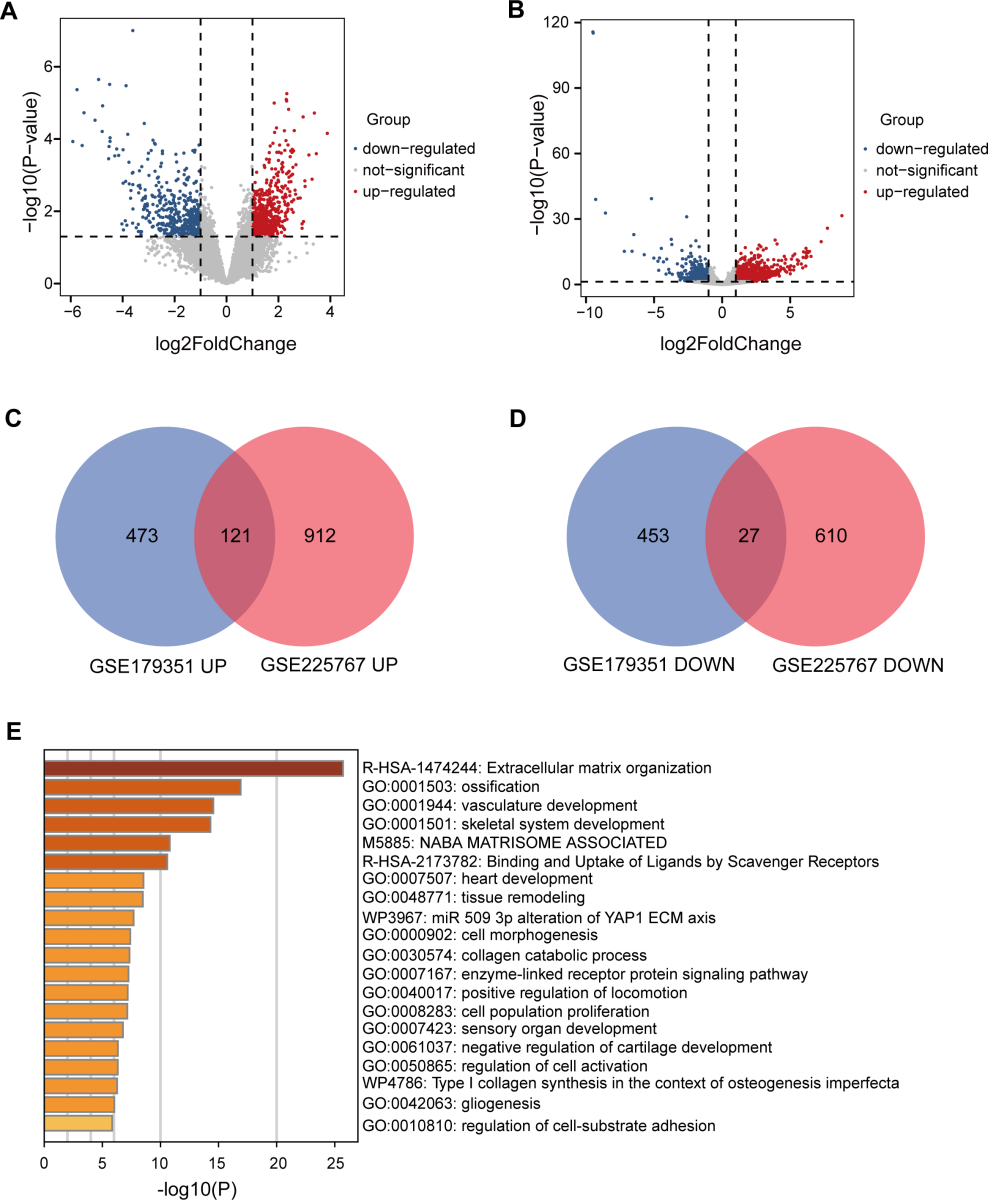
Identification and functional analysis of DEGs related to RT resistance in PAAD. **(A)** Volcano plots showing gene expression changes in PAAD tissues after RT compared to before RT in the GSE179351 datasets, with |log_2_FC| ≥1 and *p* < 0.05 set as cutoff values. In the DEGs analysis, red dots represent upregulated genes, and blue dots represent downregulated genes. **(B)** Volcano plots showing gene expression changes in PAAD tissues after RT compared to before RT in the GSE225767 datasets, with |log_2_FC| ≥1 and *p* < 0.05 set as cutoff values. In the DEGs analysis, red dots represent upregulated genes, and blue dots represent downregulated genes. **(C)** Venn diagram of upregulated genes in GSE179351 and GSE225767. **(D)** Venn diagram of downregulated genes in GSE179351 and GSE225767. **(E)** Enrichment analysis of 121 co-upregulated genes and 27 co-downregulated genes performed using the Metascape website.

To identify genes that play a key role in RT resistance, we selected two datasets, GSE179351 and GSE225767, as study objects. In these two datasets, we obtained differential genes (DEGs) in cancer tissues after RT compared with those before RT through GEO2R online analysis (**Figures 1A, B**). In the GSE179351 data set, there were 594 up-regulated genes and 480 down-regulated genes in the cancer tissues after RT compared with those before RT (**Supplementary Table S1**). In GSE225767 data, 1033 up-regulated genes and 637 down-regulated genes were found in cancer tissues after RT compared with those before RT (**Supplementary Table S2**). In DEGs analysis, the cutoff value was set as |log_2_FC| ≥1, and *p*-value <0.05. We screened 148 genes with identical expression changes in both datasets as candidate genes (**Figures 1C, D**, **Table 1**). To clarify the function of these genes, we used the Metascape tool website to conduct a republic KEGG enrichment analysis of this group of genes, which are mainly involved in the regulation of cell behavior, the development and regeneration of tissues and organs. These changes may affect cancer cell survival, invasion, and response to treatment, providing important clues to understanding the mechanism of action of RT in PAAD (**Figure 1E**).

**Table 1:** Candidate genes with consistent expression changes across both datasets.

Symbol	Change
CLMP	up
MMP2	up
KCNMA1	up
ADAM12	up
LAMA2	up
NLRP3	up
NFATC4	up
GLT8D2	up
TENM4	up
COL1A1	up
UAP1L1	up
CCN4	up
PRR16	up
GFPT2	up
ATP8B2	up
TAFA2	up
P3H1	up
HIF3A	up
ROR2	up
ISM1	up
GREB1	up
LRRC17	up
PDZRN3	up
EDNRA	up
TMEM200A	up
TBX2	up
PCDH18	up
SGIP1	up
SCD5	up
MERTK	up
CIITA	up
PAPPA	up
ADAMTS2	up
ALDH1L2	up
PRRX1	up
LEF1	up
PDGFRB	up
TSPYL2	up
COL6A2	up
NTM	up
TCF21	up
BMP8A	up
CD248	up
FKBP10	up
COL5A2	up
HLA-DOA	up
HLA-DMB	up
LZTS1	up
SLAMF8	up
NPR3	up
MAP1A	up
IL21R	up
MRC2	up
NOX4	up
NCKAP5L	up
CTSK	up
MSR1	up
CDH11	up
GLI3	up
SCARF2	up
GREM1	up
NR4A3	up
MEIS3	up
PDE1A	up
ADAMTS12	up
LAMP5	up
ITGA10	up
PLPP4	up
CCDC102B	up
PDPN	up
COL3A1	up
CD163	up
F13A1	up
FAP	up
ISLR	up
POSTN	up
PLXDC2	up
SH3PXD2B	up
SCG2	up
MMP14	up
MS4A4A	up
LRCH2	up
PPFIA2	up
CPXM1	up
DZIP1	up
CTLA4	up
TNFSF8	up
CXCL9	up
RAB3IL1	up
IRAG1	up
MFAP2	up
LOXL3	up
COL16A1	up
SPON2	up
OLFML2B	up
VCAN	up
DNAJB5	up
ITGA11	up
SULF1	up
ANGPTL2	up
NPTX1	up
COL6A3	up
BOC	up
KCND2	up
COL6A1	up
COL24A1	up
DDR2	up
SPARC	up
COL5A1	up
COL11A1	up
MLLT11	up
CTHRC1	up
MMP19	up
STMN2	up
CHPF	up
SULT1C4	up
COL1A2	up
P3H3	up
KCNE4	up
LOC107984360	up
DACT1	up
STRADB	down
RAC2	down
ANXA13	down
DMTN	down
GJB1	down
FOXA2	down
AKR1C2	down
VSIG1	down
UGT2A3	down
TNFRSF10C	down
EPHA1	down
NECTIN1	down
MAP3K21	down
ANXA10	down
ERBB3	down
HNF4A	down
GLRX5	down
SSTR1	down
CMBL	down
OSBP2	down
AKR1C3	down
GMDS	down
SULT1B1	down
TFF3	down
HBA2	down
CDHR2	down
HBA1	down

### Effect of radiation resistance gene on prognosis of patients with PAAD

**Figure 2:**
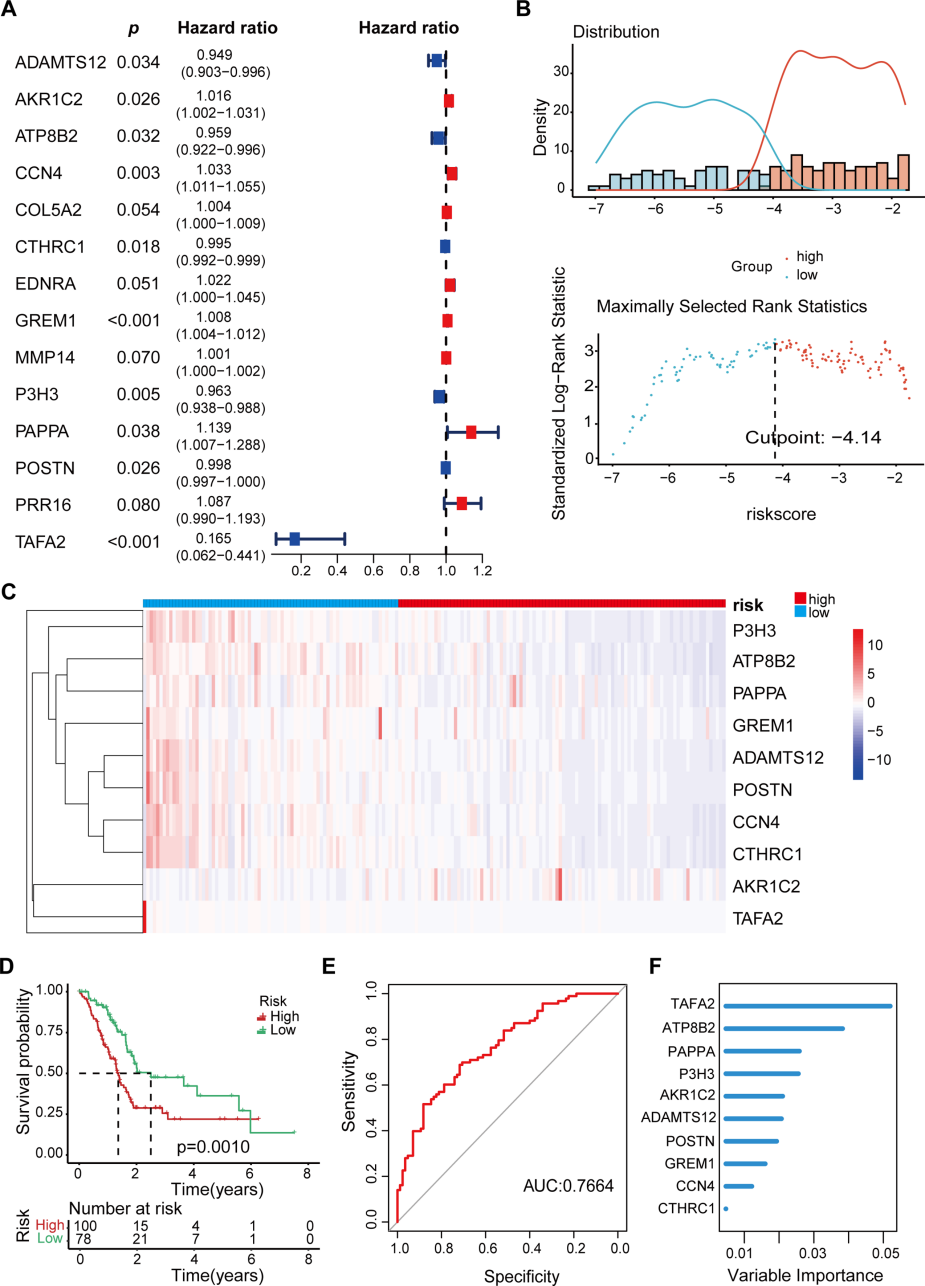
Prognostic model for PAAD Patients based on radiation-resistant genes. **(A)** Multivariate Cox regression analysis identified 10 genes associated with OS in patients with PAAD. **(B)** Optimal cutoff value of risk score determined by the “surv_cutpoint” function. 178 PAAD patients were divided into high-risk and low-risk groups. **(C)** Heat map displaying the expression levels of the 10 key genes in individual patients. **(D)** The Kaplan-Meier OS curve shows the survival differences among patients in different risk groups. **(E)** The AUC values corresponding to these gene combinations were calculated by multiple logistic regression model. The AUC value is 0.7664. **(F)** Bar plot showing the variable significance of 10 filtered genes in random forest model.

In order to investigate the effect of radiation-resistant genes on the prognosis of pancreatic cancer patients, we selected PAAD patients from the TCGA database and established univariate and multivariate Cox proportional risk regression models. Among the 148 candidate genes mentioned above, a total of 37 genes were considered to be related to patients’ overall survival (OS) by single-factor Cox regression model analysis. Of these, 26 genes are considered risk factors and 11 genes are considered protective factors. Therefore, we further included these 37 genes in multivariate Cox analysis to construct the genome associated with radiation resistance. According to the analysis results, 10 genes were screened out (*p*<0.05). Then, multivariate Cox regression coefficient and mRNA expression levels of key genes were used to establish a risk scoring formula: risk score = (-ADAMTS12*0.05286 + AKR1C2* 0.0163542-ATP8B2 *0.04237 + CCN4*0.032409 -CTHRC1*0.00454 + GREM1* 0.008157-P3H3 *0.03781 + PAPPA* 0.129872-POSTN * 0.00178-TAFA2 *1.80003). A hazard ratio greater than 1 indicates that patients with high gene expression are more likely to develop tumor progression after RT, while a hazard ratio less than 1 indicates that the gene is a protective factor (**Figure 2A**).

According to the risk score formula, 178 patients with PAAD were divided into high-risk and low-risk groups using the optimal cutoff value determined by the “surv_cutpoint” function (**Figure 2B**). The heat map identified the expression levels of 10 genes in a single patient, 100 in the high-risk group and 78 in the low-risk group (**Figure 2C**, **Table 2**). As shown in **Figure 2D**, patients with higher risk scores after RT are more likely to develop tumor progression and have a relatively shorter median survival. Specifically, when we constructed survival curves based on the expression of a single key gene, only ATP8B2, GREM1, and TAFA2 genes could obtain statistically significant results (*p*< 0.05, **Supplementary Figure S1**). However, when patients were grouped according to risk scores, the high-risk group had significantly lower survival rates at 3 and 5 years, and even beyond, compared to the low-risk group (*p* = 0.001) (**Figure 2D**). This suggests that the prognosis model is effective in distinguishing between high-risk and low-risk patients, and that high-risk patients have poorer survival outcomes. To further verify the reliability, we established a multivariate logistic regression analysis to evaluate its effectiveness in predicting tumor progression. The validated AUC value of 0.7664 indicates that the model performs well in differentiating between samples with different risk of tumor progression (**Figure 2E**). In addition, we applied machine learning methods for patient risk prediction, but on our dataset, the machine learning model had a low AUC value (**Supplementary Figure S2**). Using the random forest model, we further analyzed the contribution of the above 10 genes to the risk scoring model. The results showed that among the 148 gene candidates, *TAFA2* contributed the most to the prediction model, followed by *ATP8B2* (**Figure 2F**).

**Table 2:** Expression levels of ten key genes and risk classification in pancreatic cancer patients.

ID	Time	State	ADAMTS12	AKR1C2	ATP8B2	CCN4	CTHRC1	GREM1	P3H3	PAPPA	POSTN	TAFA2	Riskscore	Risk
TCGA-2J-AAB1	0.180822	1	11.0312	9.8524	12.7946	17.1228	134.8815	6.7831	25.4237	1.3117	115.5277	0.5221	-2.90281	high
TCGA-2J-AAB4	1.99726	0	10.9335	21.1079	18.0629	10.23	100.4886	10.479	30.4678	0.9604	46.0725	0.3656	-2.80492	high
TCGA-2J-AAB6	0.80274	1	23.5755	4.7648	9.0951	47.3707	262.571	42.8729	54.9337	4.0155	759.0189	0.0924	-3.93331	high
TCGA-2J-AAB8	0.219178	0	53.1327	3.0481	20.1468	57.6662	721.681	85.843	55.4548	6.425	772.4045	0.3016	-7.5007	low
TCGA-2J-AAB9	1.717808	1	16.7079	10.7444	16.9622	32.1665	223.3102	33.5915	26.3332	1.9408	240.261	0.3644	-2.95101	high
TCGA-2J-AABA	1.663014	1	14.6958	2.9019	24.8587	49.3591	401.2207	60.7556	43.0187	3.5387	242.1356	1.1756	-5.22387	low
TCGA-2J-AABE	1.852055	0	27.562	2.3276	14.6413	32.5273	433.2155	33.8659	41.7227	3.8785	372.0462	0.2477	-4.85827	low
TCGA-2J-AABF	1.893151	1	20.1666	20.0636	20.5365	27.1432	172.8979	12.6559	24.2278	1.9804	209.8569	0.44	-3.23468	high
TCGA-2J-AABH	3.526027	0	11.9	9.588	12.7571	11.6262	83.1506	11.5035	17.661	0.7416	210.9798	0.1846	-2.19884	high
TCGA-2J-AABI	2.654795	0	19.2703	1.8966	10.7512	36.0325	211.711	19.8967	23.2342	1.9408	474.888	0.213	-2.92925	high
TCGA-2J-AABK	1.326027	0	1.7662	6.32	8.1208	4.1619	30.5502	2.4251	18.7506	0.6726	50.2993	0.4103	-1.76787	high
TCGA-2J-AABO	1.205479	0	37.9854	4.0607	21.7695	43.0593	339.0973	62.1444	51.5613	2.5579	536.0311	0.4429	-5.86988	low
TCGA-2J-AABP	1.268493	0	50.9232	0.7125	30.8512	96.8881	382.1493	24.7142	50.731	0.9563	557.0461	0.2023	-5.53058	low
TCGA-2J-AABR	1.2	0	18.3477	54.686	29.45	33.6771	189.2273	26.239	28.1937	2.6448	316.1096	0.7288	-3.47409	high
TCGA-2J-AABT	0.873973	0	16.3477	2.527	29.9161	26.3713	127.3014	306.4978	28.4557	2.3355	349.8649	1.6028	-3.59388	high
TCGA-2J-AABU	0.758904	1	26.9933	71.0155	15.3289	34.9186	307.9644	110.4553	44.4454	2.2003	393.9212	0.1886	-2.71621	high
TCGA-2J-AABV	1.786301	1	0.5746	0.8622	1.2512	1.0831	9.8109	1.9081	7.6879	0.2294	10.5221	0.1049	-0.53163	high
TCGA-2L-AAQA	0.391781	1	21.4017	7.2762	10.431	24.2776	222.3518	35.2158	20.7029	5.6855	260.0627	0.2065	-2.26894	high
TCGA-2L-AAQE	1.873973	1	14.3402	2.3209	14.5102	16.2363	155.2664	18.2227	29.6567	2.2352	261.4931	0.5216	-3.6004	high
TCGA-2L-AAQI	0.282192	1	9.6469	4.4706	11.8486	6.0062	65.3503	2.713	19.8169	0.6233	130.339	0.2163	-2.30845	high
TCGA-2L-AAQJ	1.079452	1	11.8789	13.4556	9.3206	14.4751	135.2028	23.3718	18.7169	1.6406	226.0001	0.1555	-1.9337	high
TCGA-2L-AAQL	0.8	1	9.5673	11.7594	7.8955	12.4335	217.6106	13.9749	42.1188	0.6227	175.4138	0.2965	-3.47698	high
TCGA-2L-AAQM	3.789041	0	1.4751	0.5735	18.0476	4.2559	8.411	0.0197	130.2967	0.439	43.9492	0.3297	-6.27488	low
TCGA-3A-A9I5	4.915068	0	7.6166	27.3215	13.9704	23.5913	165.6522	13.1269	39.4826	0.7352	247.2102	0.291	-2.78951	high
TCGA-3A-A9I7	3.624658	0	33.2403	3.9609	22.7439	24.235	337.6093	178.5606	53.1392	2.7625	427.8344	0.8073	-5.81231	low
TCGA-3A-A9I9	1.736986	1	7.4349	5.2364	9.4845	9.283	116.6047	8.5128	23.077	1.1367	90.7125	0.2314	-2.17149	high
TCGA-3A-A9IB	0.613699	1	24.6137	4.8461	24.3424	28.1571	354.948	46.1839	51.3034	2.4495	644.1293	0.2846	-5.856	low
TCGA-3A-A9IC	2.021918	1	62.2107	4.5805	25.1449	49.5772	606.8547	157.0598	106.1305	3.048	823.683	0.258	-9.69435	low
TCGA-3A-A9IH	2.79726	0	11.4773	9.9969	10.9512	13.5432	149.5948	17.54	26.3944	1.0466	220.6734	0.4166	-3.00924	high
TCGA-3A-A9IJ	5.079452	0	0.0949	0.2571	16.0417	0.9397	2.8255	0.1015	42.4661	0.6893	8.9549	0.5212	-3.13239	high
TCGA-3A-A9IL	7.509589	0	1.6445	1.7948	38.3715	4.3018	27.804	3.1105	89.9137	3.0835	15.3929	1.577	-7.51035	low
TCGA-3A-A9IN	5.709589	0	1.8175	3.4466	37.7076	2.7336	21.4218	19.8523	77.7631	1.2672	86.2278	0.9531	-6.12899	low
TCGA-3A-A9IO	5.320548	0	0.5349	1.8721	31.346	1.1778	61.0787	0.2639	62.456	0.275	306.4355	0.6956	-5.6859	low
TCGA-3A-A9IR	4.224658	0	0.6866	0.109	30.1114	0.8018	3.2287	0.013	26.9177	1.5142	1.1717	44.4741	-82.1767	low
TCGA-3A-A9IS	2.734247	0	0.4267	0.2379	37.5938	1.6923	3.4814	1.1634	88.9775	0.1959	75.591	0.7118	-6.31773	low
TCGA-3A-A9IU	1.254795	1	19.1015	2.5235	12.283	27.1929	256.8873	57.2467	32.4599	1.8802	415.049	0.3412	-3.6431	high
TCGA-3A-A9IV	3.021918	0	8.4827	0.8974	42.3166	15.1989	92.508	11.5124	79.141	0.7112	94.844	0.8408	-6.64275	low
TCGA-3A-A9IX	2.841096	0	18.6749	4.7269	30.5045	37.6484	190.917	29.3938	30.7065	3.8615	316.7367	0.6507	-4.00388	high
TCGA-3A-A9IZ	0.843836	1	31.2778	10.5905	11.9092	27.661	191.1159	51.1466	32.9543	8.182	604.8928	0.1295	-3.03156	high
TCGA-3A-A9J0	2.035616	0	20.6604	6.6838	15.481	23.8394	301.4756	36.0914	39.657	1.6721	481.8008	0.4721	-4.93027	low
TCGA-3E-AAAY	6.260274	0	9.106	8.1308	20.9175	19.7518	229.6831	29.627	32.5198	0.7329	215.6603	0.3431	-3.53186	high
TCGA-3E-AAAZ	5.978082	1	24.829	3.4631	27.2713	15.3312	227.6388	236.5587	43.2802	2.7309	287.6524	1.248	-5.05885	low
TCGA-F2-6879	0.915068	1	47.2011	3.3169	19.3857	23.3572	335.1571	84.0165	23.4923	13.6582	105.3756	0.2147	-3.0308	high
TCGA-F2-6880	0.808219	0	0.1844	0.7949	2.8846	0.2474	1.573	0.533	4.2636	0.1452	1.2883	0.1005	-0.4393	high
TCGA-F2-7273	1.621918	1	29.1286	11.8308	45.5275	46.1608	249.1918	89.647	36.2994	2.9316	286.4209	0.8773	-5.26041	low
TCGA-F2-7276	0.591781	1	28.3946	19.4579	44.6835	62.631	293.4637	156.2156	29.8797	6.2466	432.7714	0.7198	-3.48896	high
TCGA-F2-A44G	0.638356	1	25.0011	1.1626	12.448	38.4685	399.4277	89.5259	30.27	3.0236	592.1273	0.3161	-4.04144	high
TCGA-F2-A44H	1.605479	0	24.221	3.4732	14.598	25.9635	368.4619	65.621	31.2733	2.4143	429.8214	0.2108	-4.15199	low
TCGA-F2-A7TX	0.260274	1	16.9084	8.0002	15.0135	20.2092	154.1891	32.1137	14.1849	4.965	200.3504	0.3752	-2.10584	high
TCGA-F2-A8YN	1.416438	0	25.4637	39.1263	12.3567	22.2432	362.1705	30.6647	27.7456	4.3855	311.6534	0.1924	-3.28411	high
TCGA-FB-A4P5	0.490411	1	12.5405	7.8017	31.8069	68.3299	250.5291	41.3549	37.9648	1.7313	107.378	0.9077	-3.50482	high
TCGA-FB-A4P6	2.10137	0	18.762	37.9152	32.2948	24.812	165.2921	30.3179	33.707	4.4075	128.939	1.1438	-4.42981	low
TCGA-FB-A545	2.005479	1	32.8683	6.2514	13.8796	30.2082	292.6111	42.053	43.1095	4.301	682.6535	0.3263	-5.10335	low
TCGA-FB-A5VM	1.364384	1	12.5201	6.2181	8.9821	24.7526	252.8665	23.3875	49.1545	0.639	255.6439	0.2981	-3.86335	high
TCGA-FB-A78T	1.027397	1	4.5984	4.4579	8.7693	12.1153	76.8433	7.1026	13.583	0.5481	88.8765	0.386	-1.73553	high
TCGA-FB-A7DR	0.967123	1	36.7244	4.8861	35.0036	29.1807	309.8342	627.5736	50.7762	4.02	729.9682	1.6057	-4.27357	low
TCGA-FB-AAPP	1.328767	1	0.6381	2.5859	1.8479	2.6667	10.0276	0.3477	4.2983	0.3755	5.3917	0.2884	-0.66851	high
TCGA-FB-AAPQ	3.09589	1	9.7365	16.245	8.1653	13.7246	114.3495	29.7461	21.1764	1.3132	222.6537	0.2548	-1.91179	high
TCGA-FB-AAPS	0.624658	0	43.0375	2.2551	37.8876	88.8121	1105.684	104.7963	127.2959	3.0216	1367.899	0.3069	-12.5392	low
TCGA-FB-AAPU	1.043836	1	4.814	0.5546	5.7634	4.4924	36.7087	3.7452	9.3369	0.322	22.0813	0.122	-1.05032	high
TCGA-FB-AAPY	2.90137	1	9.6267	39.8023	10.9375	15.8999	161.3878	13.4364	27.1201	0.7991	75.3038	0.1914	-1.82977	high
TCGA-FB-AAPZ	1.961644	0	13.8672	21.7937	18.916	24.4653	176.1353	23.2271	20.4693	1.8051	425.2593	0.3013	-2.83405	high
TCGA-FB-AAQ0	1.29589	1	8.8327	2.7049	7.1312	15.0608	124.8225	28.1822	17.407	1.9679	45.2428	0.3004	-1.5977	high
TCGA-FB-AAQ1	0.336986	1	5.5119	13.1402	6.5031	6.7812	111.4955	4.8626	17.4787	0.2381	134.2333	0.4017	-2.19085	high
TCGA-FB-AAQ2	0.419178	1	7.4793	7.2716	8.3856	9.0524	62.7969	16.2855	19.0971	0.455	139.3491	0.2039	-1.76865	high
TCGA-FB-AAQ3	0.084932	1	5.9849	5.6512	3.1769	18.2834	439.8759	15.0648	19.4475	0.7657	201.8797	0.1932	-2.98412	high
TCGA-FB-AAQ6	0.668493	1	8.3486	5.4125	6.8457	10.5883	117.232	8.0753	16.7082	0.7851	123.5482	0.1938	-1.86476	high
TCGA-H6-8124	1.073973	0	40.4416	22.8628	26.6452	39.0433	341.1871	47.3483	59.648	6.4665	667.9111	0.4336	-6.17509	low
TCGA-H6-A45N	1.087671	1	12.9986	7.1577	28.4078	32.9421	271.2982	26.7225	47.8578	1.1217	262.1196	0.9911	-5.63468	low
TCGA-H8-A6C1	1.838356	0	14.8314	7.1408	13.4688	19.2526	233.2824	13.8423	26.4208	1.833	143.9373	1.9326	-6.05648	low
TCGA-HV-A5A3	0.350685	1	10.1174	7.0432	15.403	13.9296	136.7497	22.1379	19.9559	1.0159	208.8253	0.2422	-2.49144	high
TCGA-HV-A5A4	0.635616	0	19.4813	5.2346	18.5456	25.7713	244.1671	21.6005	42.4143	1.7384	151.2125	0.3122	-4.03667	high
TCGA-HV-A5A5	0.791781	0	18.4592	29.3611	21.1914	24.778	234.0923	30.2081	31.91	0.8629	256.0975	0.3654	-3.61517	high
TCGA-HV-A5A6	5.578082	1	16.9237	1.2485	9.7969	34.9568	615.9194	84.6205	55.3224	5.5179	286.7868	0.4194	-4.90437	low
TCGA-HV-A7OL	0.690411	0	7.1964	1.8948	3.8838	8.0837	117.6904	22.6514	34.314	1.0983	125.8866	0.1784	-2.30173	high
TCGA-HV-A7OP	2.679452	0	0.2159	3.2218	0.468	0.4567	8.83	0.6364	13.2375	0.0575	0.4203	0.1121	-0.69428	high
TCGA-HV-AA8V	2.520548	0	36.78	6.6916	27.7773	46.4364	504.3325	84.0109	85.19	2.9489	673.0732	0.2016	-7.5107	low
TCGA-HV-AA8X	1.457534	1	7.7818	21.696	13.8682	10.8258	133.4712	8.9943	22.1966	0.8808	112.0269	0.4686	-2.59389	high
TCGA-HZ-7289	1.810959	1	2.8002	0.2739	16.3741	4.0231	43.8397	2.0786	22.6313	2.3827	67.8018	0.1784	-1.87714	high
TCGA-HZ-7918	2.654795	0	22.4698	2.1343	24.9443	27.2871	222.3368	30.5849	16.0002	1.2895	142.4029	2.1759	-6.69343	low
TCGA-HZ-7919	1.624658	1	32.3262	8.0942	18.3663	29.3757	266.3669	42.2455	24.7411	3.3733	419.2458	0.44	-4.30301	low
TCGA-HZ-7920	0.646575	1	13.4111	13.2684	48.7664	34.2506	114.694	107.403	40.5727	9.1679	124.6572	0.7478	-3.00437	high
TCGA-HZ-7922	0.010959	0	96.7473	16.3525	39.5474	84.1633	647.939	152.7126	41.5942	6.0605	1198.95	0.8763	-9.98762	low
TCGA-HZ-7923	0.860274	0	13.4723	12.8466	48.3133	49.2985	235.7622	67.5021	35.0725	5.9376	146.7968	1.2781	-4.58856	low
TCGA-HZ-7924	2.30137	0	3.9912	8.6522	19.7241	5.2435	14.7744	187.4463	15.3212	5.6253	62.8967	1.7888	-2.45391	high
TCGA-HZ-7925	1.682192	1	91.8433	0.3842	48.7587	159.0038	1013.261	156.4314	75.752	8.8121	1336.135	1.0669	-11.1049	low
TCGA-HZ-7926	1.419178	1	17.4351	9.5743	17.1901	24.6696	150.5928	46.1852	15.2316	3.3298	402.0882	0.6102	-2.95819	high
TCGA-HZ-8001	1.934247	0	13.3652	22.5934	26.6183	32.2109	295.8941	28.9864	65.0777	1.4778	305.1112	1.3627	-6.79299	low
TCGA-HZ-8002	1.00274	1	29.1395	36.8501	52.2118	44.1202	359.9541	42.0246	35.6701	6.1559	265.1803	1.0284	-5.88447	low
TCGA-HZ-8003	1.632877	1	13.7531	1.3036	17.9824	19.5126	103.8525	29.4089	13.8154	1.2244	81.1134	0.3004	-2.11547	high
TCGA-HZ-8005	0.328767	1	75.9184	1.6856	26.4212	90.4299	669.3878	213.7471	63.837	9.531	900.0228	0.4123	-6.99037	low
TCGA-HZ-8315	0.819178	1	28.0794	14.85	21.9766	44.9571	462.7653	57.0178	32.0889	4.2502	309.5412	0.5582	-4.56939	low
TCGA-HZ-8317	1.035616	1	11.8475	2.7531	17.6685	16.8775	99.5955	58.2314	20.5263	4.6935	166.9456	0.348	-1.85023	high
TCGA-HZ-8519	1.243836	0	11.275	4.1335	41.3584	24.8514	154.2475	41.1835	42.9727	4.3062	235.3796	0.8177	-4.79628	low
TCGA-HZ-8636	1.493151	1	33.9841	4.3147	36.6327	59.0732	445.4136	49.1065	38.9478	5.8293	585.1021	0.4541	-5.55993	low
TCGA-HZ-8637	1.416438	1	12.2635	0.8359	25.4797	42.913	241.561	23.3167	16.133	3.4129	414.2128	0.8019	-3.57743	high
TCGA-HZ-8638	0.413699	1	4.8064	11.1343	16.8412	6.4809	38.1415	193.6723	16.4889	2.0033	118.4319	0.6133	-0.8469	high
TCGA-HZ-A49G	1.808219	0	10.5865	2.0039	23.5374	17.4169	158.0284	27.1431	30.9779	0.8927	211.6409	0.5545	-3.88606	high
TCGA-HZ-A49H	1.345205	0	4.2339	6.5583	18.4504	21.5682	99.6466	19.4322	36.2898	3.0629	56.0704	0.5863	-2.62298	high
TCGA-HZ-A49I	0.843836	1	7.6905	19.4236	16.6224	17.4589	136.138	13.5145	35.3391	0.5796	178.1554	0.7067	-3.58545	high
TCGA-HZ-A4BH	0.531507	0	25.9998	12.1507	34.4609	38.8633	327.3347	68.6559	44.1457	3.0668	373.7977	0.6753	-5.45453	low
TCGA-HZ-A4BK	1.8	0	15.6738	16.2206	17.9594	9.7251	141.9134	7.5512	24.2532	2.938	148.757	1.053	-4.28759	low
TCGA-HZ-A77O	0.438356	1	11.4967	1.9379	7.9511	14.6983	98.8072	9.8489	17.1678	1.5869	213.3201	0.1122	-1.82947	high
TCGA-HZ-A77P	0.90411	0	7.8811	12.4937	32.7556	18.7651	96.9014	203.3005	40.1846	3.8436	199.9313	1.5421	-3.92558	high
TCGA-HZ-A77Q	0.090411	0	33.1942	3.6425	35.6663	90.432	635.3601	93.8356	64.1365	11.2772	822.3692	1.0055	-6.62938	low
TCGA-HZ-A8P0	0	0	14.5997	11.1768	12.2755	21.07	154.3333	18.5045	22.9555	1.7786	209.7163	0.115	-2.19336	high
TCGA-HZ-A8P1	0.019178	0	4.7909	1.4293	5.9429	5.8703	91.5047	3.7372	12.8691	0.5451	55.4274	0.7846	-2.60333	high
TCGA-HZ-A9TJ	1.652055	0	4.2046	2.3345	8.9435	5.7851	80.942	8.0759	18.0799	0.4685	87.5852	0.5026	-2.36062	high
TCGA-IB-7644	1.079452	1	39.3141	2.3882	19.0044	17.1005	288.1264	66.7542	25.8267	2.7197	195.6459	0.4722	-4.87576	low
TCGA-IB-7645	4.115068	1	29.9633	7.1397	42.0396	71.5272	397.7436	84.7767	29.6545	6.0917	696.4699	1.5647	-6.43082	low
TCGA-IB-7646	0.39726	1	69.6896	4.2302	22.0489	58.1324	655.5303	152.3739	28.178	6.4842	1444.005	0.5305	-8.14608	low
TCGA-IB-7647	1.824658	1	50.696	1.5945	32.6614	45.2403	234.3012	31.1394	38.5684	2.4947	523.0479	0.5843	-6.49807	low
TCGA-IB-7649	1.279452	1	23.7405	17.285	18.1476	14.0775	166.3787	8.6372	20.3797	1.8214	112.5899	0.3785	-3.3859	high
TCGA-IB-7651	1.652055	1	39.3937	2.7033	22.7651	45.5891	316.2014	61.635	23.8375	4.921	117.8024	0.3219	-3.5101	high
TCGA-IB-7652	3.057534	0	18.8641	5.6279	21.4016	23.8687	166.1441	6.1054	21.2495	1.4795	332.0199	0.3781	-3.6257	high
TCGA-IB-7654	1.30411	1	54.6684	1.4219	26.6631	33.9977	326.3521	56.0969	27.1429	3.3243	320.642	0.5819	-6.13165	low
TCGA-IB-7885	3.443836	0	42.0599	7.8717	21.4937	49.2182	544.8345	82.3146	47.1169	4.9276	861.5765	0.2799	-6.39146	low
TCGA-IB-7886	0.336986	1	45.9995	1.4159	28.4996	47.8598	463.2576	160.9685	22.6902	8.4053	929.0218	0.3894	-4.97574	low
TCGA-IB-7887	0.30137	1	44.3987	130.5727	15.1158	53.1813	560.3072	56.303	37.5568	6.3855	189.7601	0.2931	-2.67044	high
TCGA-IB-7888	3.649315	1	16.9224	8.7919	55.2823	68.6547	215.0607	16.0162	37.1199	5.6778	105.154	1.0976	-4.54333	low
TCGA-IB-7889	1.317808	1	8.867	2.143	14.5237	13.7111	174.4669	15.8448	16.9396	0.7134	135.6862	0.2899	-2.57902	high
TCGA-IB-7890	1.638356	1	79.9872	4.1478	35.6251	54.9389	626.5741	123.8991	66.2262	6.6537	1502.137	0.2967	-10.5704	low
TCGA-IB-7891	2.50137	1	30.204	3.6029	26.263	36.8652	259.6315	46.4514	25.461	2.1449	345.7105	0.3273	-4.14436	low
TCGA-IB-7893	0.320548	1	128.3399	14.8155	42.0405	106.1429	1117.708	535.7758	73.0246	5.6852	2686.869	0.3106	-12.9505	low
TCGA-IB-7897	1.331507	1	18.9629	21.6283	71.6983	50.144	155.697	37.2164	35.7137	9.5212	212.4967	1.6388	-5.90685	low
TCGA-IB-8126	1.265753	0	3.7512	3.1716	12.8515	13.3582	67.9622	28.9425	9.893	1.2308	68.5594	0.5904	-1.72957	high
TCGA-IB-8127	1.430137	0	40.0627	8.286	21.86	39.9471	467.0047	68.8855	29.1193	4.474	445.5046	0.4486	-5.2932	low
TCGA-IB-A5SO	1	1	20.507	2.5952	28.3161	43.1559	329.1426	53.1743	51.2865	1.997	191.1492	0.6823	-5.15222	low
TCGA-IB-A5SP	1.320548	0	2.6816	4.7878	5.418	2.9802	43.2195	2.4815	18.5766	0.2593	27.6288	0.1657	-1.38868	high
TCGA-IB-A5SQ	0.6	1	46.7278	1.5673	32.0113	76.1978	650.0031	73.7161	62.1988	3.4312	819.7536	0.5553	-8.04646	low
TCGA-IB-A5SS	1.260274	1	69.7132	7.8747	25.5743	77.3972	582.6837	125.5602	67.524	5.9846	1271.82	0.1851	-8.12527	low
TCGA-IB-A5ST	1.739726	0	18.4889	4.8009	36.0072	40.8723	358.7042	11.5314	37.8803	1.9948	528.5901	0.8412	-6.26278	low
TCGA-IB-A6UF	1.824658	0	6.6175	6.8173	8.8551	7.5997	112.2675	16.1237	17.4925	0.7177	148.2833	0.3787	-2.2593	high
TCGA-IB-A6UG	0.112329	1	6.6366	6.6646	9.8941	6.8933	89.5887	13.8496	10.7669	0.8852	94.8983	0.1448	-1.45322	high
TCGA-IB-A7LX	0.684932	1	12.9427	63.2522	8.6976	12.4945	166.9181	18.2675	19.8261	2.0161	80.3198	0.357	-1.49587	high
TCGA-IB-A7M4	1.323288	0	7.1707	39.7781	9.8773	13.6296	190.2009	29.0187	25.3024	0.897	168.1421	0.2091	-1.84831	high
TCGA-IB-AAUM	0.021918	0	4.3035	2.3579	10.0127	8.6756	70.765	5.5145	12.6028	1.5618	75.9446	0.4775	-1.87677	high
TCGA-IB-AAUN	0.394521	1	24.3762	2.8189	11.0892	25.5204	198.929	28.214	23.0989	4.2012	174.436	0.3266	-2.78466	high
TCGA-IB-AAUO	0.654795	1	7.7276	12.6988	6.2623	15.5268	109.9648	23.0765	22.0396	1.5647	134.224	0.3299	-1.73695	high
TCGA-IB-AAUP	1.180822	0	23.2653	31.3986	43.6136	37.9431	347.6323	51.2086	36.1077	2.6896	377.1014	1.3695	-6.64786	low
TCGA-IB-AAUQ	0.50137	1	17.7355	60.8696	18.607	24.493	218.5418	34.5212	65.823	1.118	138.2383	0.2688	-3.72128	high
TCGA-IB-AAUR	0.926027	0	16.4571	5.4403	53.7154	37.1109	217.5444	35.2199	38.9775	5.1608	213.9105	0.8407	-5.25245	low
TCGA-IB-AAUS	0.616438	0	32.8268	4.6006	29.377	102.6023	681.3167	94.3207	69.2312	5.7987	623.3245	0.7061	-6.14946	low
TCGA-IB-AAUT	0.786301	0	15.3271	24.3241	24.4176	16.2564	230.7331	51.6565	36.3078	1.1417	107.1861	0.4438	-3.76104	high
TCGA-IB-AAUU	0.671233	0	10.7432	17.9239	12.9665	14.3005	179.3833	21.0768	19.4678	1.0014	164.4403	2.1844	-5.83416	low
TCGA-IB-AAUV	1.106849	0	26.2004	6.2881	62.0636	107.6792	609.8911	160.1793	96.1066	3.1397	767.7111	3.1641	-12.1731	low
TCGA-IB-AAUW	0.630137	1	2.9007	19.1176	28.1984	12.0011	38.3903	11.1943	23.0832	3.0837	57.5629	0.6422	-2.4603	high
TCGA-L1-A7W4	0.761644	1	16.6721	0.9772	9.5713	44.4105	185.6045	63.5494	21.1316	3.6174	435.9294	0.3299	-1.85466	high
TCGA-LB-A7SX	1.076712	1	2.9115	24.9726	5.7865	6.6086	49.6459	13.4806	8.8098	1.515	28.2326	0.2552	-0.53801	high
TCGA-LB-A8F3	1.038356	0	6.0186	1.7771	7.7964	7.3818	172.3833	13.803	32.746	0.3386	111.8639	0.2641	-2.91927	high
TCGA-LB-A9Q5	0.857534	1	6.8361	2.3322	8.1619	7.7835	79.7018	7.2605	11.8207	0.4926	175.3017	0.2979	-1.9506	high
TCGA-M8-A5N4	1.6	0	23.8785	0.7714	16.7828	46.0965	375.8754	80.9991	30.1812	3.3447	789.3646	0.4691	-4.4686	low
TCGA-OE-A75W	0.731507	1	13.2425	8.7665	6.7587	21.3996	272.7934	27.7136	56.6804	1.8494	121.7226	0.2062	-3.65336	high
TCGA-PZ-A5RE	1.287671	1	16.8636	3.4684	10.6457	29.7048	347.4718	113.3712	52.8789	2.5306	106.4778	0.1699	-3.14284	high
TCGA-Q3-A5QY	1.139726	0	8.9032	13.8426	31.9545	26.7706	151.7169	16.0264	26.8841	1.4466	189.7652	0.7769	-3.85366	high
TCGA-Q3-AA2A	0.260274	0	6.5386	2.5832	7.2023	10.41	125.0782	6.372	21.246	0.7872	134.6995	0.2138	-2.11293	high
TCGA-RB-A7B8	1.276712	1	34.7373	18.4647	18.2725	36.3413	347.8167	33.9842	39.8325	5.4679	267.9989	0.2242	-4.10975	high
TCGA-RB-AA9M	0.783562	0	18.182	13.0244	23.9235	16.0949	170.7542	15.8164	35.109	2.0677	181.912	0.5511	-4.26135	low
TCGA-RL-AAAS	0.024658	0	10.3344	26.2175	22.4158	19.7806	205.7121	37.5074	57.3474	0.9161	204.8091	0.4197	-4.22397	low
TCGA-S4-A8RM	2.019178	0	4.9254	6.3576	10.7697	13.944	133.1727	5.9059	11.1438	1.0874	58.6572	0.3548	-1.74072	high
TCGA-S4-A8RO	1.438356	0	10.8106	5.8928	52.6167	14.0359	133.0328	12.1905	23.3605	0.7643	186.0594	0.1479	-4.13565	low
TCGA-S4-A8RP	1.923288	1	20.2103	1.6156	20.6681	29.3239	267.7289	23.668	38.5388	3.112	294.5077	0.4265	-4.33499	low
TCGA-US-A774	1.90411	1	37.3532	9.7553	24.3006	47.8875	382.1325	52.9042	38.1996	5.8888	450.109	0.574	-5.11033	low
TCGA-US-A776	3.331507	0	0.6262	3.2995	5.1498	2.1456	15.561	4.8979	72.3381	0.1902	7.607	0.2316	-3.29956	high
TCGA-US-A779	1.4	1	2.6203	4.8973	4.4084	1.6164	29.6404	1.7068	13.6565	0.1939	19.9476	0.314	-1.40544	high
TCGA-US-A77E	1.178082	1	26.9139	9.4119	17.4652	43.3647	494.994	93.397	55.0583	2.5352	438.1139	0.1957	-4.97425	low
TCGA-US-A77G	0.032877	1	1.3829	5.9146	5.1517	3.312	23.1204	1.8594	6.8251	0.2146	19.0575	0.2609	-0.9109	high
TCGA-US-A77J	1.556164	1	11.3683	10.739	23.5169	32.4058	169.4924	28.2459	47.006	1.932	88.7726	0.7082	-3.87023	high
TCGA-XD-AAUG	1.150685	0	43.2121	9.8194	45.3235	92.4952	604.0959	125.7172	89.8697	2.2895	251.5683	2.5195	-10.8486	low
TCGA-XD-AAUH	1.082192	0	10.9565	7.2167	43.4566	23.7009	119.0274	27.8667	43.5933	3.103	128.9576	0.9865	-5.09816	low
TCGA-XD-AAUI	1.00274	1	12.9832	9.1868	19.276	24.4304	181.8525	22.8924	31.4864	1.4673	246.8304	0.3387	-3.24906	high
TCGA-XD-AAUL	1.364384	0	32.0187	23.4568	23.2341	48.2837	496.3852	73.4731	48.7542	2.7784	596.9153	0.4717	-5.77748	low
TCGA-XN-A8T3	2.605479	0	28.1143	8.5288	21.1569	27.2931	320.056	13.9124	37.5754	2.0779	609.6431	0.4512	-5.74629	low
TCGA-XN-A8T5	1.972603	0	12.3593	4.5326	47.0847	29.4411	191.2754	75.4539	47.0946	6.3156	101.7147	0.9982	-4.81173	low
TCGA-YB-A89D	0.958904	0	37.0532	7.3276	27.6661	51.1657	486.856	68.4849	69.4271	2.913	467.9056	0.2213	-6.48324	low
TCGA-YH-A8SY	1.063014	0	50.1298	12.4444	25.8574	84.7246	788.753	182.5825	96.9103	4.3286	784.5356	0.0498	-7.47708	low
TCGA-YY-A8LH	5.523288	0	1.5164	10.8523	3.665	2.8325	40.9966	2.117	13.5343	0.4854	31.641	0.9003	-2.2607	high
TCGA-Z5-AAPL	1.279452	0	5.8782	4.7564	40.7341	23.1342	58.8741	33.555	13.2255	4.1332	114.6118	1.0135	-3.19431	high

Next, we verified the 10 genes selected above to verify their relationship with radiation resistance in pancreatic cancer. The results showed that *TAFA2* and *POSTN* were significantly elevated in IR-resistant pancreatic cancer cells (**Supplementary Figure S3**), suggesting that TAFA2 may play an important role in radiotherapy resistance of pancreatic cancer.

### Cross-dataset validation and clinical association analysis of PAAD score models

**Figure 3:**
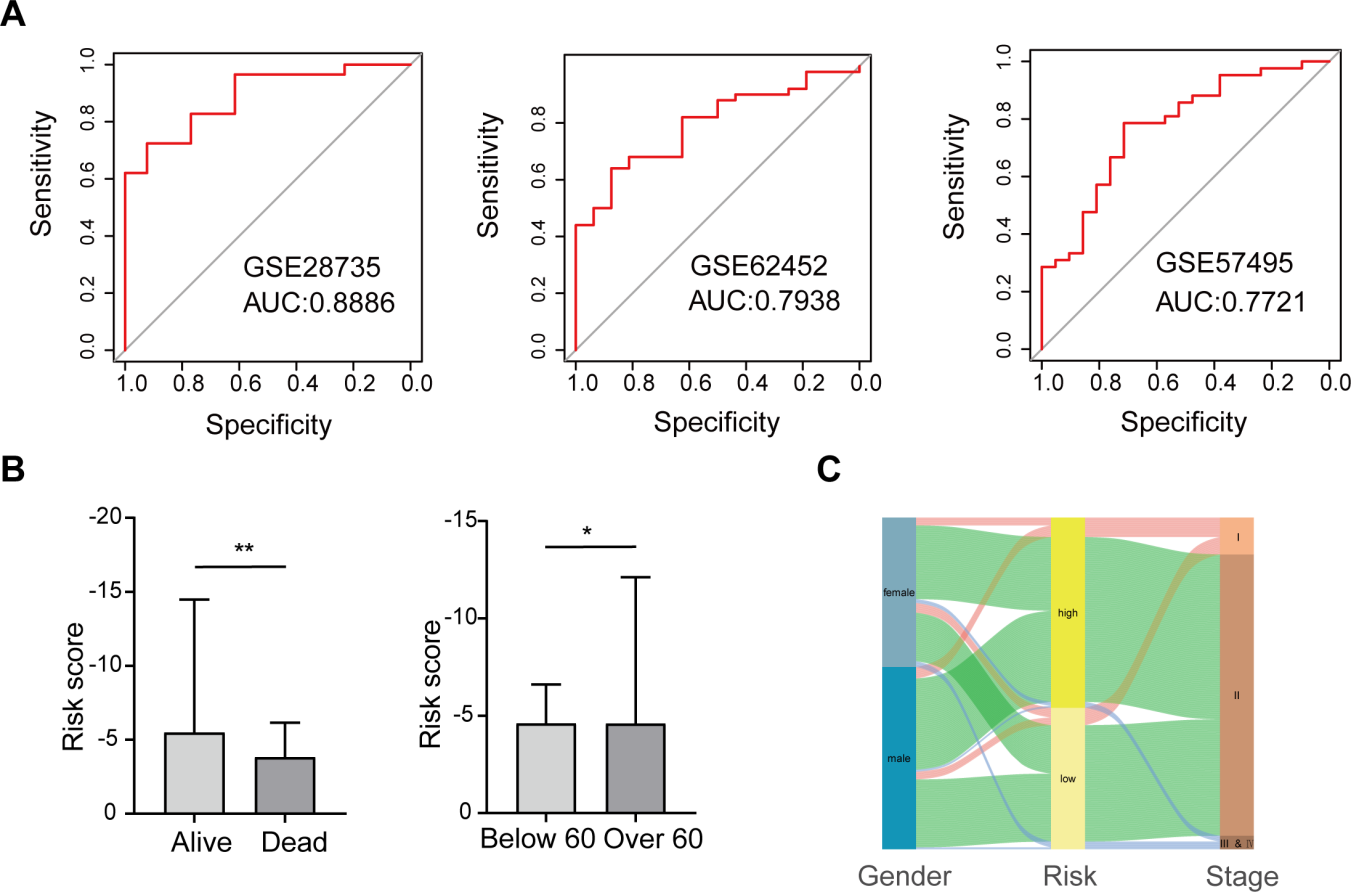
Validation of the scoring model’s predictive performance and clinical relevance. **(A)** ROC curves for three GEO datasets. **(B)** Risk scores compared by survival status and age group. **(C)** Sankey diagram depicting relationships among gender, risk group, and cancer stage. *p < 0.05; **p < 0.01.

To more fully validate the predictive power of the scoring model, we downloaded and analyzed three publicly available pancreatic cancer GEO datasets (GSE28735, GSE62452, and GSE57495). In each dataset, we calculated the AUC value via the ROC curve to assess the accuracy of the model’s prediction of patient risk. The results showed that all datasets had AUC values higher than 0.77, with the GSE28735 dataset having the highest AUC value of 0.8886, indicating that the scoring model has stable and high predictive performance across multiple datasets (**Figure 3A**), supporting its potential to be widely used in diverse pancreatic cancer patient populations.

Further, we explored the association between the risk score of the scoring model and the clinical characteristics of patients, aiming to analyze the clinical significance of the score. The study found that the risk score was not significantly associated with gender or pathological stage, suggesting consistent applicability of the score to patients of different genders and stages (**Supplementary Figure S4**). However, there was a significant correlation between risk scores and patients’ survival status and age. Specifically, patients whose survival status was death had a significantly higher risk score than those who survived, suggesting that this score may be a powerful indicator of prognosis. In addition, patients older than 60 years had significantly higher risk scores than those younger than 60 years, a finding that may reflect a more aggressive or progressive course of disease in older patients (**Figure 3B**).

We also mapped the relationship between gender, high-low risk groups, and cancer stage to visualize the interaction patterns between these variables (**Figure 3C**). In **Figure 3C**, the distribution of patients of different genders in high and low risk groups and cancer stages is shown in the form of Sankey charts. Although there was no significant association between gender and risk score, we could observe differences in disease stage among patients in different risk groups. Such visualization not only helps to understand the relationship between variables, but also provides a reference for the development of further individualized treatment strategies.

In summary, this study confirmed the strong predictive ability of the scoring model in pancreatic cancer patients through external validation of multiple GEO datasets, and supported the clinical application potential of the model through correlation analysis with clinical characteristics. This validation method based on multiple data sets not only enhances the robustness of the model, but also lays a foundation for its popularization in clinical practice.

### Enrichment analysis of DEGs in high and low-risk group

**Figure 4:**
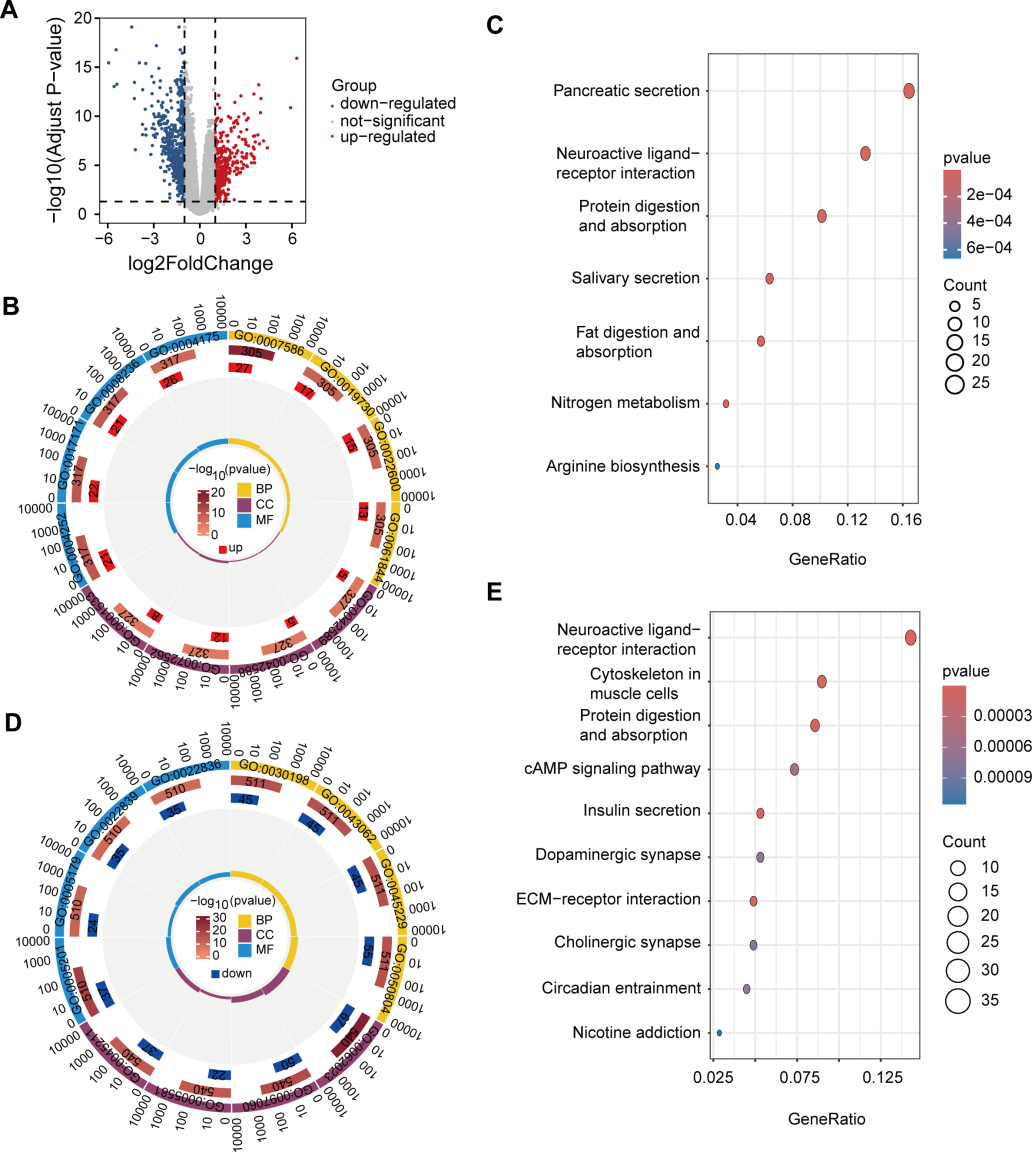
DEGs and functional enrichment analysis in high- and low-risk groups. **(A)** Volcano plot of 933 DEGs with 348 upregulated (red) and 585 downregulated (blue) genes. **(B)** GO enrichment of upregulated genes. **(C)** KEGG enrichment of upregulated genes in pathways such as pancreatic secretion, neuroactive ligand-receptor interaction, and protein digestion, supporting tumor growth and metabolic demands in high-risk patients. **(D)** GO enrichment of downregulated genes. **(E)** KEGG enrichment of downregulated genes in pathways like neuroactive signaling, cytoskeletal organization, and metabolism, indicating reduced proliferation and migration potential in low-risk patients.

Based on the matched tumor RNA-seq data from PAAD patients, we identified 933 DEGs (*p*.adj < 0.05 and |log_2_FC| ≥ 1) between the high-risk and low-risk groups, including 348 up-regulated genes and 585 down-regulated genes (**Figure 4A**). Next, we performed GO and KEGG enrichment analysis for these differential genes.

The results of GO enrichment analysis showed that the up-regulated genes were mainly enriched in GO terms associated with immune response, cell differentiation, and digestion, processes that may be involved in tumor development and changes in the immune microenvironment of PAAD (**Figure 4B**). GO enrichment results of down-regulated genes showed that these genes were mainly related to the tissue and structural components of the extracellular matrix, basic enzyme activity, and molecular binding activity, suggesting that tumor progression may be slower in low-risk patients, and tissue remodeling and signaling activities may be less active (**Figure 4D**).

The results of KEGG enrichment analysis showed that up-regulated genes were mainly enriched in pancreatic secretion, neuroactive ligand-receptor interactions, and protein digestion and absorption pathways (**Figure 4C**). The enrichment of these pathways suggests that patients at high risk of PAAD exhibit active biological characteristics in digestion, metabolism and nerve signaling, providing support for the growth, metabolic needs and microenvironment regulation of PAAD cells, thereby promoting the invasion and metastasis of cancer cells.

In contrast, KEGG enrichment of down-regulated genes showed that these genes were mainly concentrated in pathways such as neuroactive ligand-receptor interactions, cytoskeleton of muscle cells, protein digestion and absorption, and insulin secretion (**Figure 4E**). These pathways show lower activity in low-risk PAAD patients, particularly in pathways related to nerve signaling, cytoskeleton, metabolism, and extracellular matrix. Downregulation of these pathways may limit tumor cell proliferation, migration, and nutrient acquisition, thereby slowing tumor aggressiveness and progression.

### Immune and tumor microenvironment differences in high- and low-risk PAAD patients

After enrichment analysis of differentially expressed genes in high and low risk groups of PAAD, we found that up-regulated genes were significantly enriched in immune response, cell differentiation and digestion. Among them, GO terms related to immune response stand out, suggesting that there may be important molecular and cellular changes in the immune microenvironment in high-risk PAAD patients. Given that the immune system plays a key role in the occurrence, development and prognosis of tumors, it is necessary to further explore the clinical significance and biological characteristics of these immune-related genes. Therefore, our next step is to focus on screening for immune-related genes in these differential genes and performing survival analyses on them to assess their impact on the prognosis of patients with PAAD.

**Figure 5:**
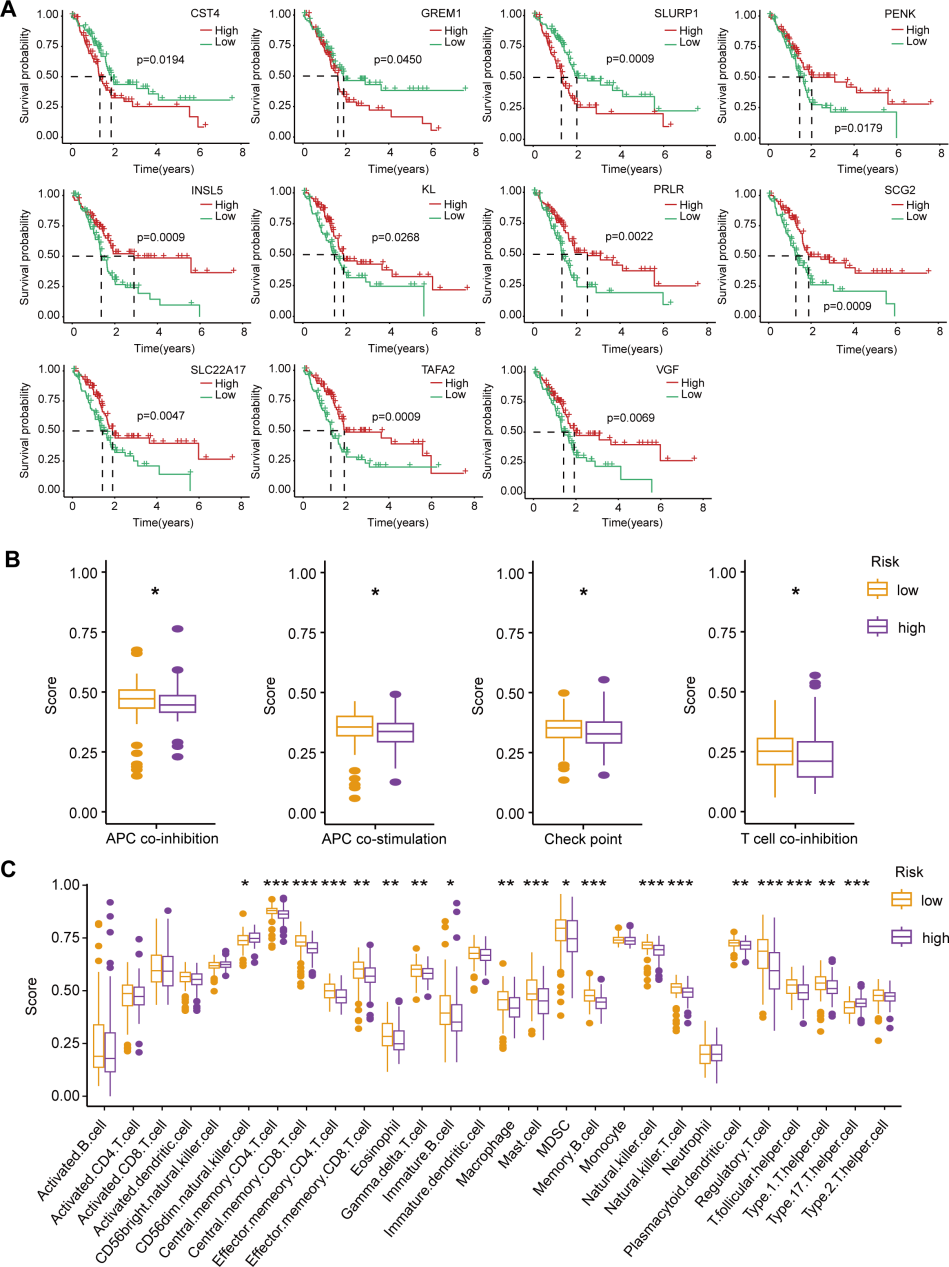
Survival analysis of immune-related DEGs, immune function, and immune infiltration differences between high-risk and low-risk PAAD patients. **(A)** Kaplan-Meier survival curves for 11 immune-related DEGs significantly associated with OS in PAAD patients. **(B)** Comparison of immune function scores between high-risk (purple) and low-risk (orange) groups. **(C)** Immune cell infiltration scores comparing high-risk and low-risk groups. *p < 0.05; **p < 0.01; ***p < 0.001.

Based on the ImmPort database, we identified 113 immune-related DEGs among the differentially expressed genes in the high-low risk group. Through univariate Cox regression analysis and Kaplan-Meier survival analysis, we further screened 11 immune-associated DEGs that were significantly associated with OS in PAAD patients. Among them, CST4, GREM1 and SLURP1 were favorable factors, while PENK, INSL5, KL, PRLR, SCG2, SLC22A17, TAFA2 and VGF were risk factors (**Figure 5A**).

In addition, to fully understand the role of immunity in PAAD progression, we also analyzed differences in immune function and immune infiltration between high and low risk groups. Immune function analysis showed that in the low-risk group, APC co-inhibition, APC co-stimulation, immune checkpoint, and T cell co-inhibition were highly active (**Figure 5B**). The high activity of these immune functions may indicate that the immune system of patients in the low-risk group achieves a balance between anti-tumor response and autoimmune protection. Enhanced APC and T cell inhibitory signaling, as well as regulation of immune checkpoints, help maintain the homeostasis of the immune microenvironment, thereby inhibiting tumor progression.

Immune infiltration analysis revealed a significant increase in CD56 dim natural killer cells and type 17 T helper cells in the high-risk group, potentially leading to a stronger pro-inflammatory response and an immune escape environment that accelerates malignant progression of tumors. In contrast, in the low-risk group, central memory CD4/CD8 T cells, effector memory CD4/CD8 T cells, eosinophils, gamma delta T cells, immature B cells, macrophages, mast cells, myeloid suppressor cells (MDSC), memory B cells, natural killer cells, natural killer T cells, plasmacytoid dendritic cells, regulatory T cells, and T follicular helpers) cell and type 1 T helper cells were more infiltrated (**Figure 5C**). These enhanced infiltrations of memory and effector immune cells, along with moderate immunomodulatory mechanisms, help suppress tumor progression and maintain anti-tumor immune surveillance.

Overall, the high and low risk groups showed significant differences in immune function and immune cell infiltration. In the high-risk group, an increase in pro-inflammatory immune cells may lead to a more aggressive tumor microenvironment; In the low-risk group, moderate immune balance and diversified immune cell infiltration may contribute to tumor suppression. These results provide important clues for understanding the immune microenvironment of PAAD and its impact on patient prognosis, and may provide a basis for personalized immunotherapy for PAAD patients.

### Analysis of individualized treatment for PAAD

**Figure 6:**
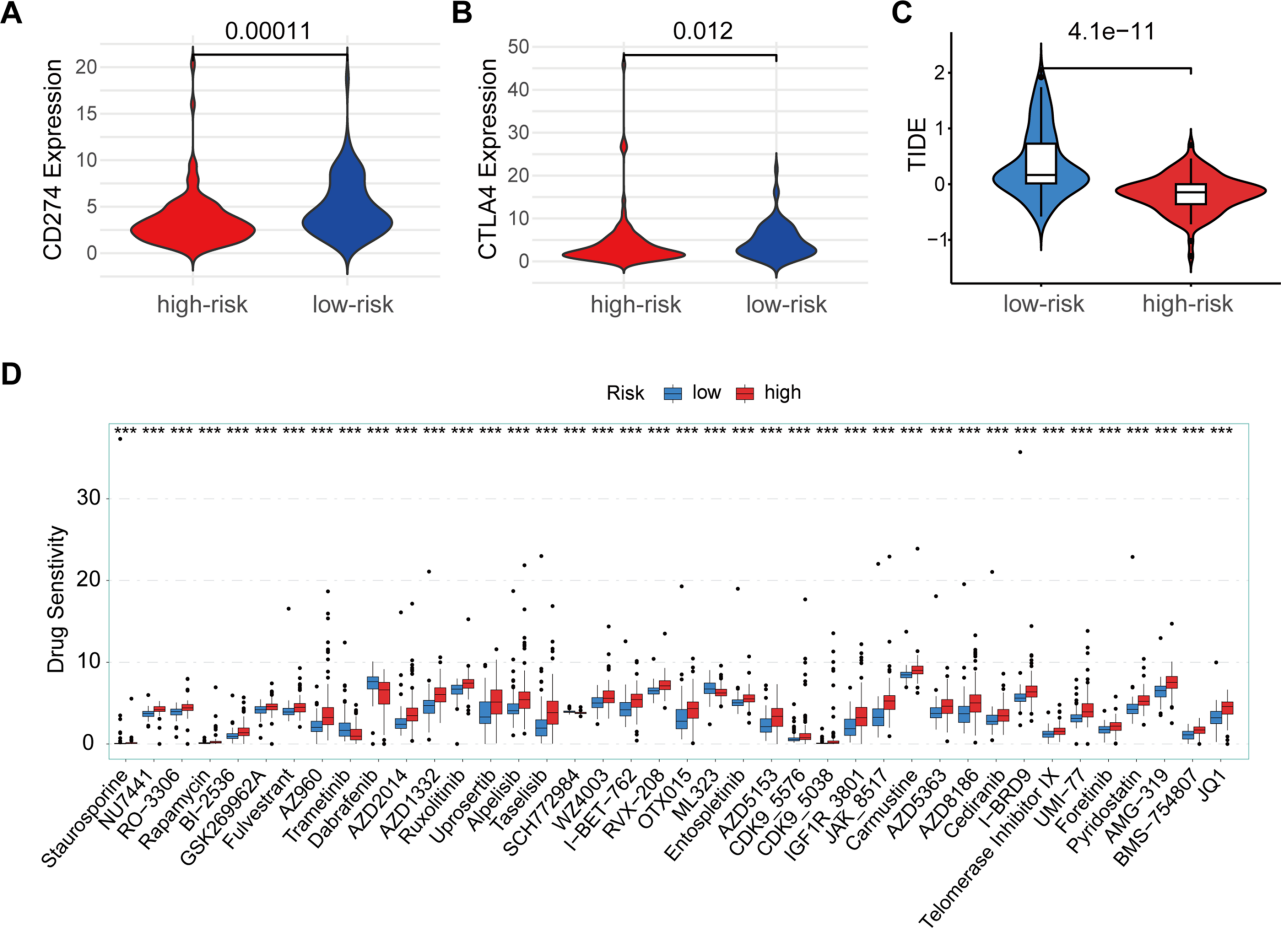
Immune checkpoint and drug sensitivity analysis. **(A)** Comparison of CD274 expression between high-risk (red) and low-risk (blue) groups. **(B)** Comparison of CTLA4 expression between high-risk (red) and low-risk (blue) groups. **(C)** TIDE scores between low-risk (blue) and high-risk (red) groups. **(D)** Drug sensitivity analysis between high-risk (red) and low-risk (blue) groups across multiple anti-cancer drugs. ***p < 0.001.

Many studies have shown that patients with high expression levels of CD274 or CTLA4 may benefit more from immunotherapy (12, 13). Based on the above analysis of immune characteristics and tumor microenvironment in the high and low risk group of PAAD patients, we further investigated the differences of CD274 and CTLA4, two important immune checkpoint molecules, between the high and low risk groups. We observed significant differences in the expression of CD274 and CTLA4 in the high-low risk group, and the expression of CD274 and CTLA4 in the low-risk group was higher than that in the high-risk group (**Figures 6A, B**). This suggests that the low-risk group may have a relatively mild immune microenvironment compared to the high-risk group, rather than an overactivated pro-inflammatory environment. The high expression of CD274 and CTLA4 can reduce the overreaction of the immune system, thereby inhibiting the release of pro-inflammatory cytokines, and may help delay the malignant progression of tumors. At the same time, we also made TIDE predictions. The results showed that TIDE scores were higher in the low-risk group than in the high-risk group, with higher TIDE scores generally indicating a stronger immune escape capacity and a poorer response to immunotherapy (**Figure 6C**). However, the high TIDE score in patients in the low-risk group may be mainly caused by high expression of CD274 and CTLA4, and the expression of this immune checkpoint is targetable. Therefore, a high TIDE score in the low-risk group is not necessarily a marker of a malignant prognosis, but may instead mean that these patients are more sensitive to CD274 or CTLA4 inhibitors.

In addition, we assessed differences in sensitivity to multiple antineoplastic drugs in high-low risk groups (**Figure 6D**). The results showed that patients in the high-risk group had a high sensitivity to Trametinib, Dabrafenib, SCH772984, ML323, indicating that patients in the high-risk group were more sensitive to these chemotherapy agents, suggesting that PAAD patients in the high-risk group may benefit more from these drugs. In contrast, high-risk patients were insensitive to drugs such as Staurosporine, NU7441, O-3306, Rapamycin, BI-2536, GSK269962A, Fuverastine, AZ960, AZD2014, AZD1332, Rusolitinib, Uprosertib, Alpelisib, Taselisib, WA4003, I-BET-762, RVX-208, OTX015, Entospletinib, AZD5153, CDK9-5576, CDK9-5038, IGF1R-3801, JAK-8517, Carmustine, AZD5363, AZD8186, Cediranib, I-BRD9, telomerase Inhibitor IX, Uni-77, Foretinib, Pyridostatin, AMG-319, BMS-754807, and JQ1. The difference in sensitivity between different drugs further highlights the significant differences in tumor microenvironment and biology between the high and low risk groups, and also suggests potential directions in individualized treatment options.

Overall, there were significant differences in immune checkpoint gene expression, immune escape ability, drug sensitivity, and immune function and infiltrating cells in the high and low risk groups for PAAD. These differences not only deepen our understanding of the immune microenvironment of PAAD, but also provide a valuable basis for personalized immunotherapy. Future studies should further explore the practical application value of these immune features in patients with PAAD, with a view to optimizing the treatment of patients and improving the treatment effect and survival rate of PAAD.

## Discussion

This study offers valuable insights into the mechanisms underlying RT resistance and the immune microenvironment of PAAD, as well as the implications of these factors for personalized treatment strategies. By constructing a robust prognostic scoring model, validated across multiple GEO datasets, we identified a clear distinction in survival outcomes between high-risk and low-risk PAAD patients. The risk model, developed based on differential gene expression profiles in response to RT, effectively stratifies patients and demonstrates strong predictive performance, with higher risk scores correlating with poorer survival outcomes.

Pancreatic cancer is not the most common type of cancer, but it is of great concern because of its high fatality rate (14, 15). To improve prognostic survival for pancreatic cancer, there is an urgent need to find strong biomarkers for patients. In this study, we constructed a reliable RT prognosis scoring model based on a publicly available GEO dataset. In the TCGA training session, we confirmed the clinical value of this model. In addition, our RT prognosis scoring model was demonstrated to have reliable predictive power in three separate datasets (GEO28735, GEO62452, and GEO57495). To confirm the association between RT and genes associated with RT prognosis, we are conducting further functional studies. PAAD patients were grouped by a scoring model, and this combination of genes helps predict patients’ RT outcomes and may serve as an indicator for assessing RT response.

In clinical applications, RT is the primary treatment for PAAD, but its efficacy is limited by the heterogeneity of patient response. By dividing patients into those who respond well to RT and those who do not, side effects can be reduced and the recurrence of surviving cancer cells can be inhibited, a promising treatment strategy. However, RT showed a heterogeneous response in different PAAD patients, suggesting that patients’ immune microenvironment may influence their sensitivity to RT. Our findings highlight the significant heterogeneity in tumor biology and immune response between high- and low-risk PAAD groups. In high-risk patients, the up-regulation of genes associated with immune response and cell differentiation suggests an immune microenvironment that may facilitate tumor progression and immune escape. This pro-inflammatory environment, indicated by increased infiltration of CD56 dim natural killer cells and type 17 T helper cells. The increase of NK cells and Th17 cells in tumor tissue tends to release more pro-inflammatory factors, further promoting the inflammatory response (16, 17). This inflammatory state may make tumors more aggressive, as inflammation plays an important role in cancer progression, often associated with cancer cell proliferation, invasion, angiogenesis, and so on (18, 19). This pro-inflammatory environment is consistent with the aggressive nature of pancreatic cancer, which is often resistant to conventional treatments, including radiation (20, 21). Conversely, low-risk patients showed enriched immune functions such as APC co-stimulation, immune checkpoint, and T-cell co-inhibition. High score of co-stimulation and co-inhibitory in APC indicates increased activity in antigen presentation and immune response regulation (22). This means that the immune system of these patients is more inclined to engage in anti-tumor activity and may be more likely to recognize and respond to tumor antigens. High immune checkpoint score is often part of immune escape, but in the low-risk group of patients, this may be because the immune system is still effectively trying to regulate and attack tumor cells, and this regulation can be maintained with a low disease burden (23). The high score of T cell co-inhibition may indicate that although T cells are activated, their activity is suppressed to a certain extent due to the existence of regulatory mechanisms (22, 24). This may be the case in the low-risk group to balance the anti-tumor immune response and prevent an excessive immune response that leads to tissue damage. This suggesting a more balanced immune microenvironment capable of anti-tumor response without excessive inflammation (21, 25).

The differences in immune cell infiltration and immune checkpoint gene expression between high- and low-risk groups underscore the need for tailored immunotherapy strategies. *CD274*, also known as PD-L1 (Programmed Death-Ligand 1), is an important immune checkpoint molecule in immune system regulation (26). It plays a key role in the immune escape mechanism of tumors. *CD274*/PD-L1 is expressed in many types of tumors and inhibits T cell activity through interaction with its receptor, PD-1, thereby helping tumor cells evade host immune surveillance (27, 28). Tumor cells often overexpress PD-L1 to evade attack by the immune system (29). This immune escape mechanism helps tumor cells survive and spread in the body, making PD-L1 expression levels associated with poorer prognosis in many tumor types (30). PD-L1 expression is generally not limited to tumor cells, but can also be expressed in some immune cells in the tumor microenvironment, such as macrophages and dendritic cells (31–33). This expression plays an auxiliary role in regulating the immunosuppressive state of the tumor microenvironment, thereby reducing the immune attack of the entire microenvironment on tumor cells (34). CTLA4 is another key immune checkpoint molecule. CTLA4 is mainly expressed in activated T cells and regulatory T cells (35–38). When T cells are activated by antigen stimulation, CTLA4 binds to its ligands B7-1 (*CD80*) and B7-2 (*CD86*) to transmit inhibitory signals, thereby reducing T cell activation and proliferation (39). This process helps prevent the immune system from overreacting and protects the body’s tissues from excessive inflammation and autoimmune damage (40).

The higher expression of CD274 and CTLA4 in low-risk patients suggests that they may benefit more from immune checkpoint inhibitors, as these molecules help regulate immune response and prevent the release of excessive pro-inflammatory cytokines, thereby potentially limiting tumor progression. Interestingly, the TIDE score analysis further supports this possibility, indicating that while low-risk patients show a higher immune escape potential, their immune profile could still be targeted with CD274 or CTLA4 inhibitors.

Additionally, our analysis of drug sensitivity differences across risk groups provides practical implications for chemotherapy choices. High-risk PAAD patients demonstrated higher sensitivity to drugs like Trametinib, Dabrafenib, SCH772984, and ML323, suggesting that these agents could be prioritized in treatment plans for these patients. On the other hand, the insensitivity of high-risk patients to a range of other drugs further underscores the need for more effective, targeted therapies that consider the unique tumor microenvironmental features of each risk group.

This study presents a framework for personalized treatment in PAAD, with specific emphasis on understanding immune and biological characteristics to guide therapy. By integrating gene expression data, immune characteristics, and drug response profiles, this study not only provides a basis for tailored therapy but also contributes to the broader goal of improving outcomes for PAAD patients. Future research should aim to validate these findings in larger, prospective cohorts and further investigate the potential of using immune-related biomarkers to predict responses to immunotherapy, with the ultimate objective of optimizing treatment and improving survival rates in PAAD.

## Data Availability

The original contributions presented in the study are included in the article/**Supplementary Material**. Further inquiries can be directed to the corresponding authors.

## Supplementary material

Supplementary Figure 1 Kaplan-Meier survival curves for PAAD patients based on the expression of key genes. **(A)** Survival probability for patients stratified by ATP8B2 expression. Patients in the high-risk group (red line) showed significantly lower survival probability compared to those in the low-risk group (green line), with a *p*-value of 0.0069. **(B)** Survival probability for patients stratified by GREM1 expression. High-risk patients (red line) exhibited lower survival probability than low-risk patients (green line), with a *p*-value of 0.0450. **(C)** Survival probability for patients stratified by TAFA2 expression. The high-risk group (red line) demonstrated a significantly reduced survival probability relative to the low-risk group (green line), with a *p*-value of 0.0009.

Supplementary Figure 2 Comparison of risk scores in PAAD patients based on gender and tumor stage. **(A)** Distribution of risk scores between male and female patients. There was no significant difference in risk scores between genders (ns indicates non-significant). **(B)** Distribution of risk scores between patients with early-stage (T1 & T2) and advanced-stage (T3 & T4) tumors. No significant difference in risk scores was observed between these stages (ns indicates non-significant).

Supplementary Figure 3 Expression of 10 risk genes in radiation-resistant pancreatic cancer cells. **(A)** The relative expression of *POSTN*. **(B)** The relative expression of *TAFA2*. **(C)** The relative expression of *ADAMTS12*. **(D)** The relative expression of *AKR1C2*. **(E)** The relative expression of *ATP8B2*. **(F)** The relative expression of *CCN4*. **(G)** The relative expression of *CTHRC1*. **(H)** The relative expression of *GREM1*. **(I)** The relative expression of *P3H3*. **(J)** The relative expression of *PAPPA*. IR: Ionizing radiation. **p* < 0.05; ***p* < 0.01.

Supplementary Figure 4 Comparison of risk scores in PAAD patients based on gender and tumor stage. **(A)** Distribution of risk scores between male and female patients. There was no significant difference in risk scores between genders (ns indicates non-significant). **(B)** Distribution of risk scores between patients with early-stage (T1 & T2) and advanced-stage (T3 & T4) tumors. No significant difference in risk scores was observed between these stages (ns indicates non-significant).

Supplementary Table 1 Differential expression of GSE179351 gene before and after radiotherapy.

Supplementary Table 2 Differential expression of GSE179351 gene before and after radiotherapy.
