# Supplementary figures and images for: Exploring radiation resistance-related genes in pancreatic cancer and their impact on patient prognosis and treatment
Source: Front Immunol. 2025 Mar 3;16:1524798. doi: 10.3389/fimmu.2025.1524798 (PMC11914796; doi:10.3389/fimmu.2025.1524798)

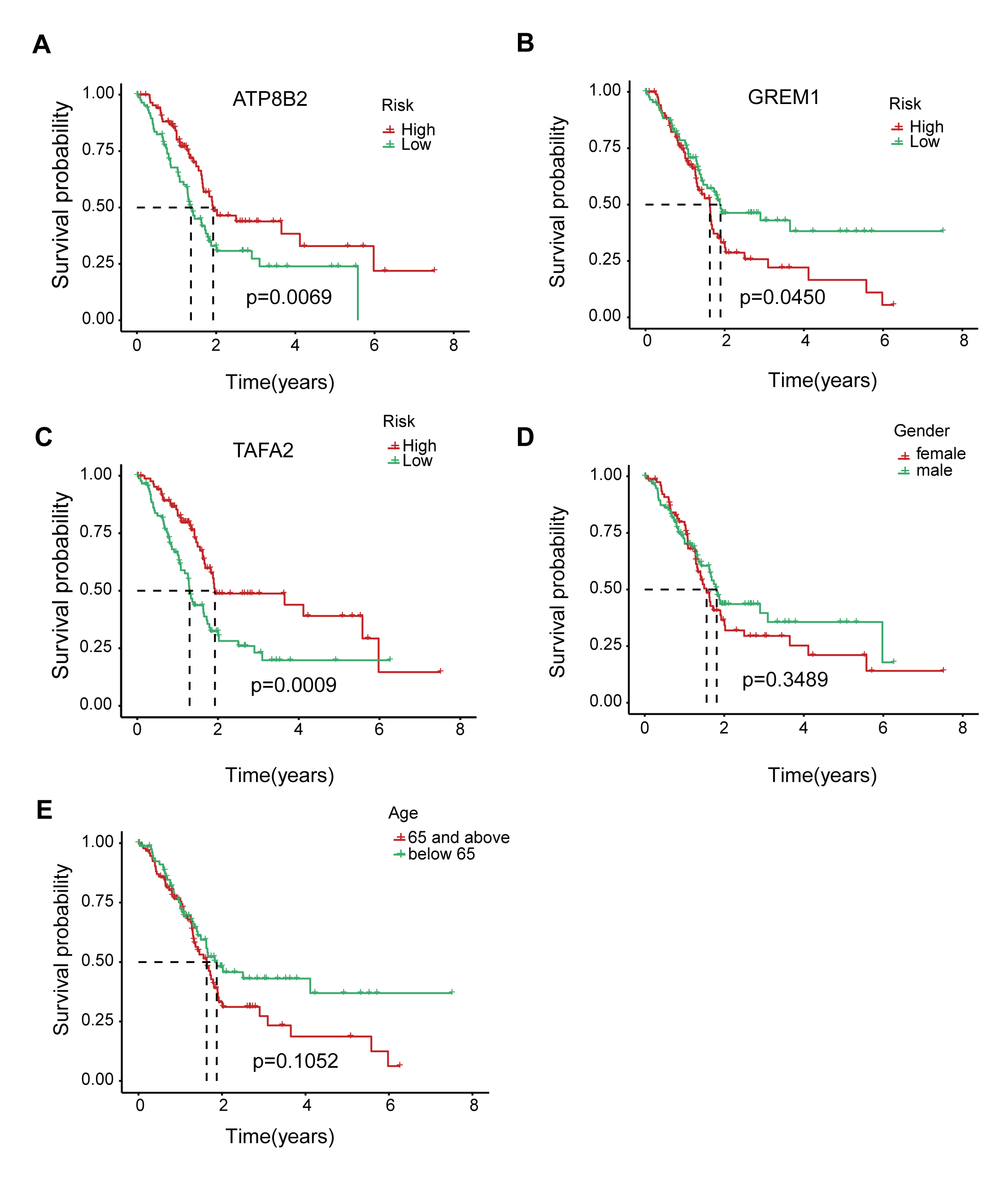

Supplement: Supplementary Figure 1 — Kaplan-Meier survival curves for PAAD patients based on the expression of key genes. (A) Survival probability for patients stratified by ATP8B2 expression. Patients in the high-risk group (red line) showed significantly lower survival probability compared to those in the low-risk group (green line), with a p-value of 0.0069. (B) Survival probability for patients stratified by GREM1 expression. High-risk patients (red line) exhibited lower survival probability than low-risk patients (green line), with a p-value of 0.0450. (C) Survival probability for patients stratified by TAFA2 expression. The high-risk group (red line) demonstrated a significantly reduced survival probability relative to the low-risk group (green line), with a p-value of 0.0009. [file Image1.tif]

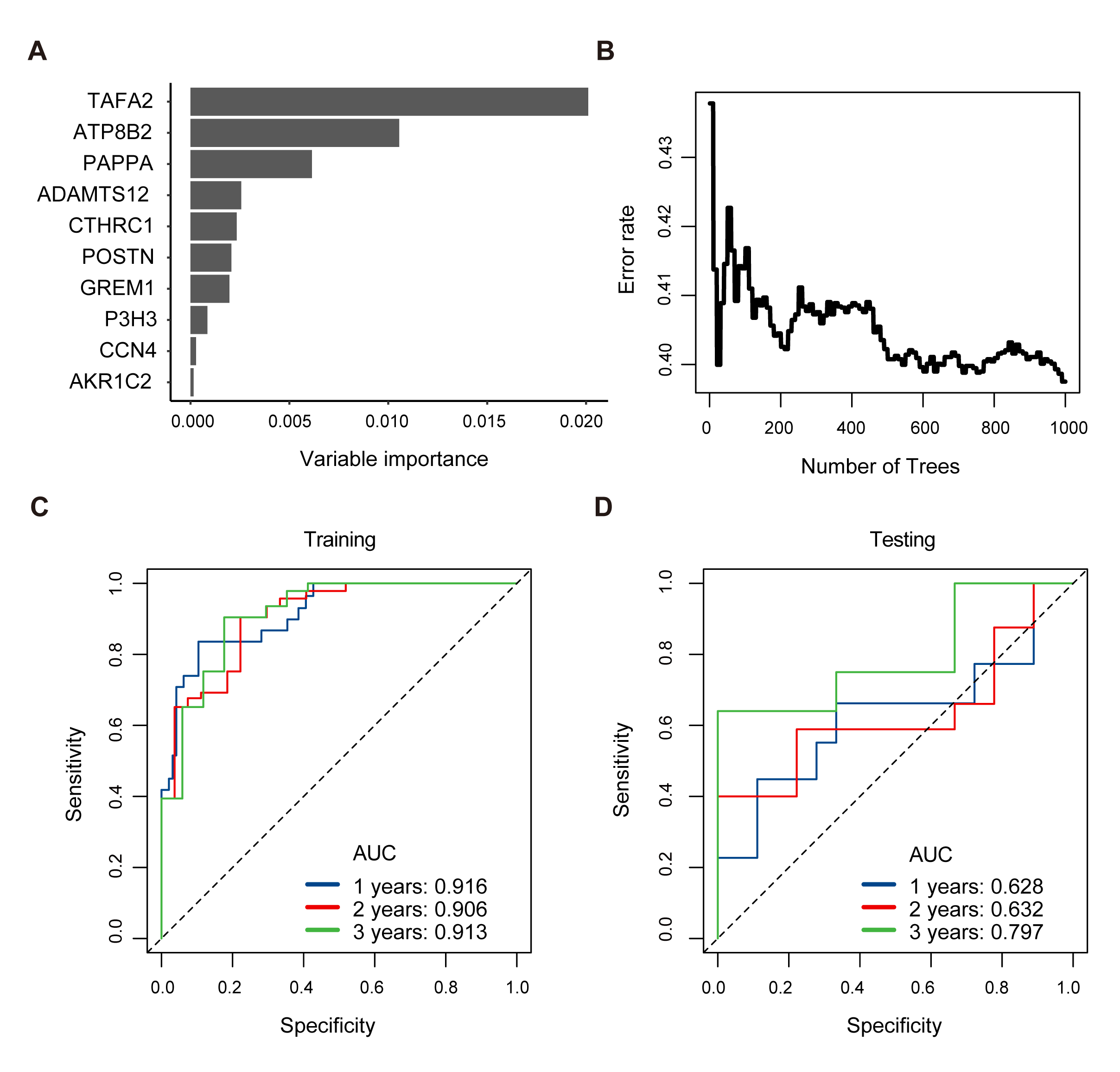

Supplement: Supplementary Figure 2 — Comparison of risk scores in PAAD patients based on gender and tumor stage. (A) Distribution of risk scores between male and female patients. There was no significant difference in risk scores between genders (ns indicates non-significant). (B) Distribution of risk scores between patients with early-stage (T1 & T2) and advanced-stage (T3 & T4) tumors. No significant difference in risk scores was observed between these stages (ns indicates non-significant). [file Image2.tif]

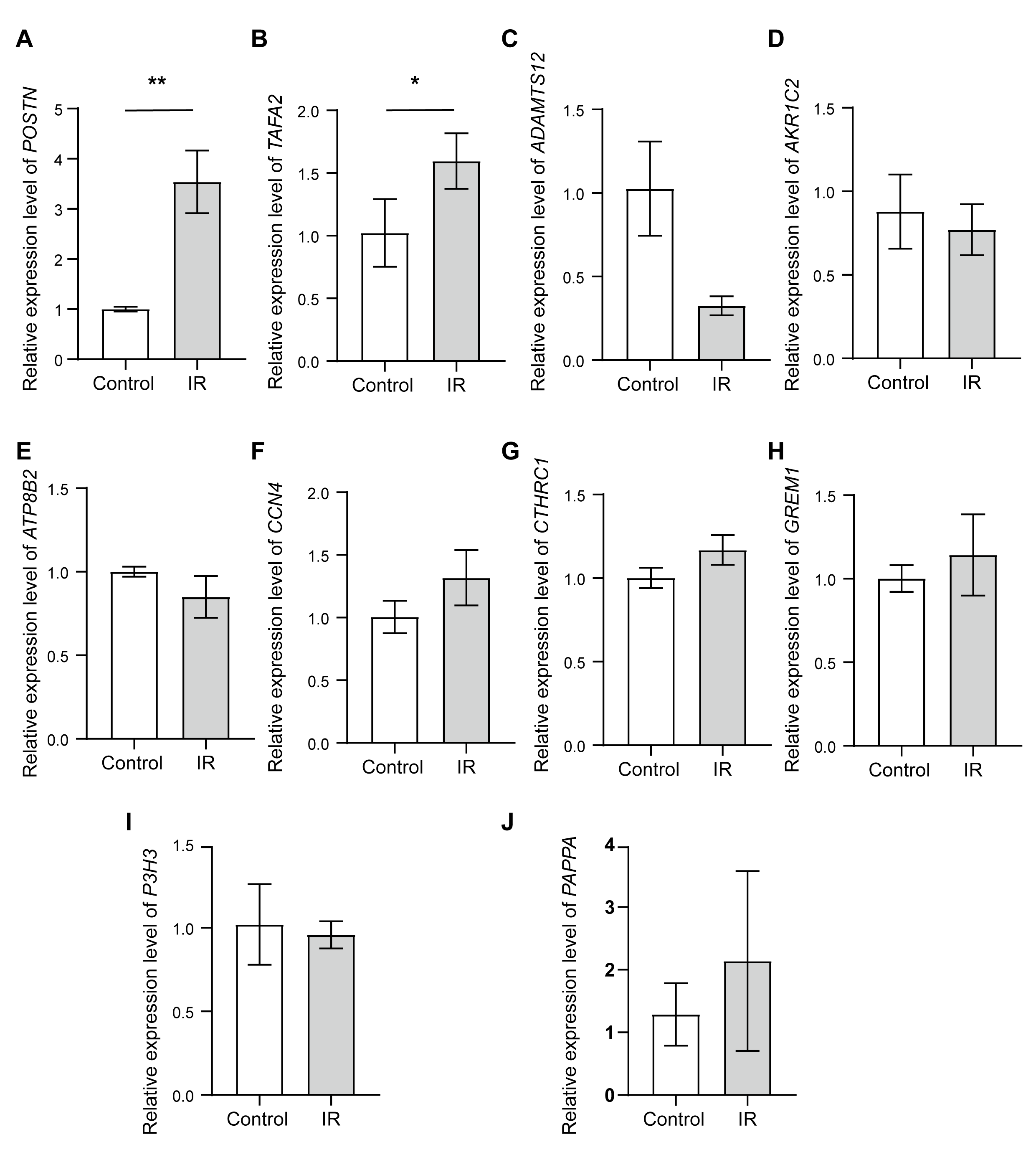

Supplement: Supplementary Figure 3 — Expression of 10 risk genes in radiation-resistant pancreatic cancer cells. (A) The relative expression of POSTN. (B) The relative expression of TAFA2. (C) The relative expression of ADAMTS12. (D) The relative expression of AKR1C2. (E) The relative expression of ATP8B2. (F) The relative expression of CCN4. (G) The relative expression of CTHRC1. (H) The relative expression of GREM1. (I) The relative expression of P3H3. (J) The relative expression of PAPPA. IR: Ionizing radiation. *p < 0.05; **p < 0.01. [file Image3.tif]

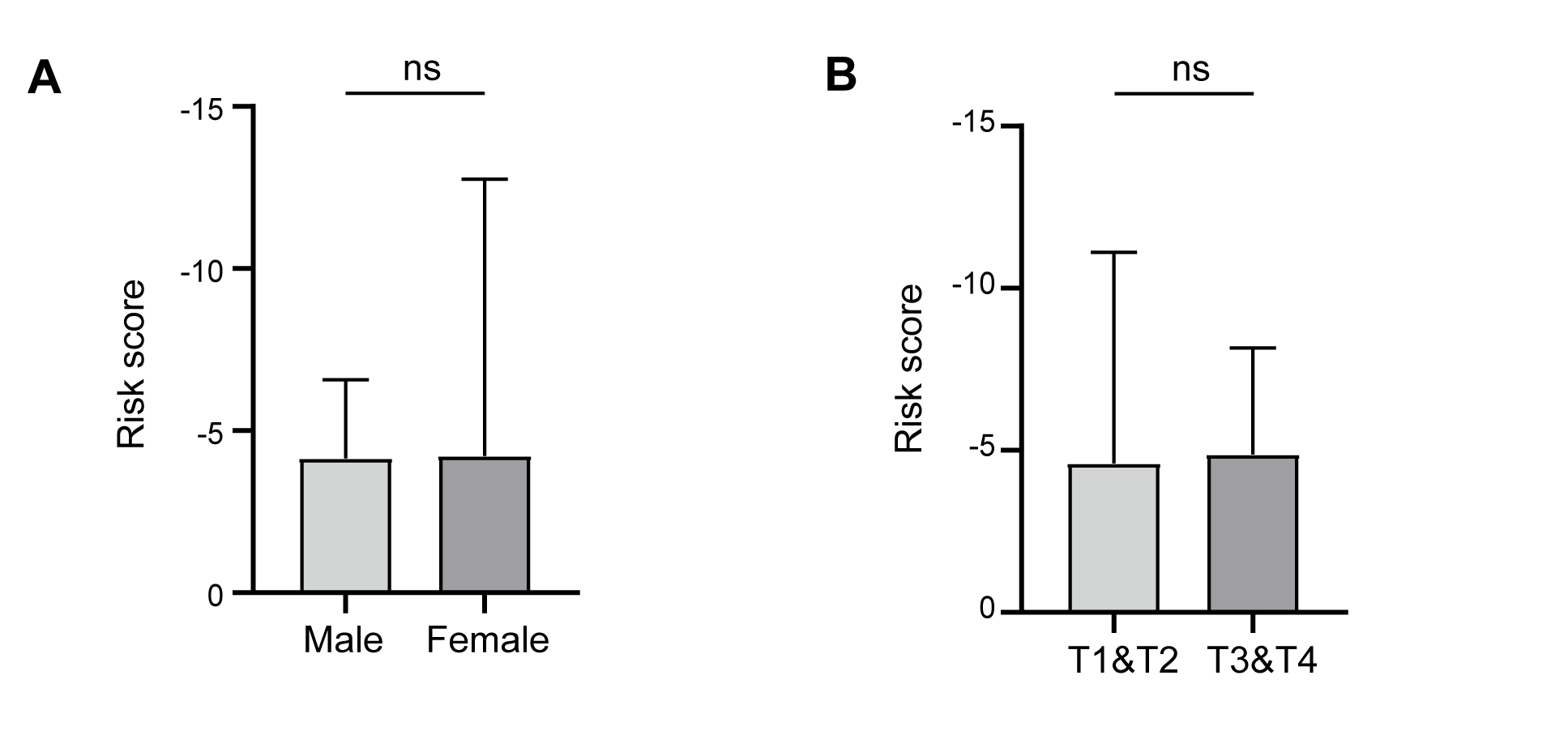

Supplement: Supplementary Figure 4 — Comparison of risk scores in PAAD patients based on gender and tumor stage. (A) Distribution of risk scores between male and female patients. There was no significant difference in risk scores between genders (ns indicates non-significant). (B) Distribution of risk scores between patients with early-stage (T1 & T2) and advanced-stage (T3 & T4) tumors. No significant difference in risk scores was observed between these stages (ns indicates non-significant). [file Image4.tif]
